# The entry of unclosed autophagosomes into vacuoles and its physiological relevance

**DOI:** 10.1371/journal.pgen.1010431

**Published:** 2022-10-13

**Authors:** Zulin Wu, Haiqian Xu, Pei Wang, Ling Liu, Juan Cai, Yun Chen, Xiaomin Zhao, Xia You, Junze Liu, Xiangrui Guo, Tingting Xie, Jiajie Feng, Fan Zhou, Rui Li, Zhiping Xie, Yanhong Xue, Chuanhai Fu, Yongheng Liang

**Affiliations:** 1 College of Life Sciences, Key Laboratory of Agricultural Environmental Microbiology of Ministry of Agriculture and Rural Affairs, Nanjing Agricultural University, Nanjing, China; 2 National Laboratory of Biomacromolecules, CAS Center for Excellence in Biomacromolecules, Institute of Biophysics, Chinese Academy of Sciences, Beijing, China; 3 University of Chinese Academy of Sciences, Beijing, China; 4 Ministry of Education Key Laboratory for Membrane-less Organelles & Cellular Dynamics, CAS Center for Excellence in Molecular Cell Sciences, Hefei National Laboratory for Physical Sciences at the Microscale, School of Life Sciences, Division of Life Sciences and Medicine, University of Science and Technology of China, Hefei, China; 5 State Key Laboratory of Microbial Metabolism, School of Life Sciences and Technology, Shanghai Jiao Tong University, Shanghai, China; The University of North Carolina at Chapel Hill, UNITED STATES

## Abstract

It is widely stated in the literature that closed mature autophagosomes (APs) fuse with lysosomes/vacuoles during macroautophagy/autophagy. Previously, we showed that unclosed APs accumulated as clusters outside vacuoles in Vps21/Rab5 and ESCRT mutants after a short period of nitrogen starvation. However, the fate of such unclosed APs remains unclear. In this study, we used a combination of cellular and biochemical approaches to show that unclosed double-membrane APs entered vacuoles and formed unclosed single-membrane autophagic bodies after prolonged nitrogen starvation or rapamycin treatment. Vacuolar hydrolases, vacuolar transport chaperon (VTC) proteins, Ypt7, and Vam3 were all involved in the entry of unclosed double-membrane APs into vacuoles in Vps21-mutant cells. Overexpression of the vacuolar hydrolases, Pep4 or Prb1, or depletion of most VTC proteins promoted the entry of unclosed APs into vacuoles in Vps21-mutant cells, whereas depletion of Pep4 and/or Prb1 delayed the entry into vacuoles. In contrast to the complete infertility of diploid cells of typical autophagy mutants, diploid cells of Vps21 mutant progressed through meiosis to sporulation, benefiting from the entry of unclosed APs into vacuoles after prolonged nitrogen starvation. Overall, these data represent a new observation that unclosed double-membrane APs can enter vacuoles after prolonged autophagy induction, most likely as a survival strategy.

## Author summary

Normal autophagy is critical for cellular homeostasis, whereas defective autophagy is closely linked to human disease development and progression. The model organism yeast has been greatly contributing to basic and applied researches of autophagy. Previously, we found that unclosed double-membrane APs accumulated as clusters outside vacuoles in haploid yeast Vps21/Rab5 and ESCRT mutants after a short period of nitrogen starvation. If these APs could not enter vacuoles to be degraded, the autophagy in these mutants would be completely interrupted to be defective. However, we found herein that the unclosed double-membrane APs in Vps21-mutant cells entered vacuoles to be degraded after prolonged autophagy induction or to be unclosed single-membrane autophagic bodies if further lacking vacuolar hydrolases. When we cultured diploid Vps21-mutant cells in a sporulation medium, their unclosed APs also entered vacuoles to be degraded, so that these diploid mutant cells could progress through meiosis to sporulation as diploid wild-type cells, not to be complete infertile as diploid cells of typical autophagy mutants. VTC proteins, Ypt7, and Vam3 were all important for the entry of unclosed double-membrane APs into vacuoles. Facilitating the entry of unclosed APs into lysosomes for degradation through autophagy may serve as a remedy strategy in autophagosome-closure-impaired diseases.

## Introduction

Autophagy involves multiple steps, including initiation, phagophore expansion, closure, fusion, and cargo degradation in lysosomes/vacuoles [1,2]. Impaired or defective autophagy is closely linked to disease development and progression [3]. It has been widely described in the literature that the outer membrane of closed mature double-membrane autophagosomes (APs) fuses with the lysosome/vacuole membrane. Then, the closed AP only containing the inner membrane is thought to be released into the lysosome/vacuole to form a single-membrane autophagic body (AB), which is further degraded by hydrolases and recycled [4,5]. However, the above-described fusion step has not been clearly or thoroughly demonstrated through experimentation. Previous transmission electron microscope (TEM) data showed that, in yeast, the double-membrane AP structures contacted the vacuole membrane and occasionally formed a continuous membrane between the AP membrane and the vacuole membrane [6]. However, the double membrane of APs and single membrane of ABs were not clearly shown in that study due to the resolution limit of conventional TEM. Thus, it was unclear whether only the outer membrane of APs (but not the inner membrane or both membranes) dynamically fused with lysosome/vacuole membranes in live cells. Fusion of the outer membrane of closed, mature double-membrane APs with lysosome/vacuole membranes requires additional experimental support, given that the double membrane of APs and single membrane of ABs can be observed clearly and dynamically. In addition, only closed APs were proposed to fuse with vacuoles/lysosomes as closed APs accumulated in yeast *ypt7Δ* or *vam3Δ* cells or the corresponding mammalian AP-lysosome fusion mutant lines [7–9], although no experimental evidence excludes the possibility that unclosed APs can enter vacuoles. Therefore, it remains unknown whether unclosed APs (i.e., phagophores) can fuse with lysosome/vacuole membranes to deliver unclosed APs into lysosomes/vacuoles for degradation.

Previously, we found that AP closure was impaired in yeast Vps21- or ESCRT (the endosomal sorting complex required for transport)-mutant cells, in which unclosed APs accumulated as clusters around vacuole membranes (mostly outside the vacuoles) shortly after autophagy was induced (i.e., nitrogen starvation for 2 h or treatment with rapamycin for 4 h) [10,11]. However, it remains to be determined whether the unclosed autophagosome clusters (APCs) can enter vacuoles for degradation.

In our previous studies [10,11], we observed a few interesting phenotypes in Vps21- or ESCRT-mutant cells after nitrogen starvation: 1) not all mutant cells in the same culture displayed APC accumulation, 2) older cultures before nitrogen starvation were less likely to accumulate APCs, 3) a higher percentage of mutant cells accumulated APCs when they lacked the vacuolar hydrolase Pep4, and 4) APCs were occasionally seen inside vacuoles. As the expression levels of yeast vacuolar proteases change in a growth-stage-dependent manner and peak when yeast cells approach the stationary phase [12,13], it is possible that the older cultures of Vps21- or ESCRT-mutant cells expressed higher levels of vacuolar proteases. Increased levels of vacuolar proteases might promote the entry of APCs into vacuoles. Conversely, the absence of vacuolar proteases in Vps21- or ESCRT-mutant cells might inhibit the entry of APCs into vacuoles. Our observations suggest that the entry of unclosed APCs into vacuoles might depend on the levels of vacuolar proteases, contrary to the paradigm that only closed, mature APs can enter lysosomes/vacuoles through fusion.

In this study, we induced the accumulation of unclosed APCs outside vacuoles in Vps21- or ESCRT-mutant cells, with or without vacuolar proteases, and then assessed the fate of the unclosed APCs after a prolonged period of nitrogen starvation or rapamycin treatment. Using cellular and biochemical methods, we demonstrated that unclosed double-membrane APCs can ultimately enter vacuoles and become unclosed single-membrane ABs after prolonged nitrogen starvation. Furthermore, we found that the vacuolar proteases Pep4 and Prb1, several VTC proteins, the Rab GTPase Ypt7, and the SNARE Vam3 played important roles in affecting the entry of unclosed double-membrane APCs into vacuoles. This process contributed to meiosis and sporulation in diploid Vps21-mutant cells. Thus, we discovered an unexpected phenomenon that unclosed double-membrane APCs in yeast could enter vacuoles after a prolonged period of autophagy induction. This process prevents autophagy from being completely interruption and restores the cells to some level of normal physiology, such that sporulating diploid Vps21-mutant cells can survive under induced stress.

## Results

### Growth-stage- and hydrolase-dependent accumulation of unclosed APCs in *vps21Δ* cells after a short period of autophagy induction

**Fig 1:**
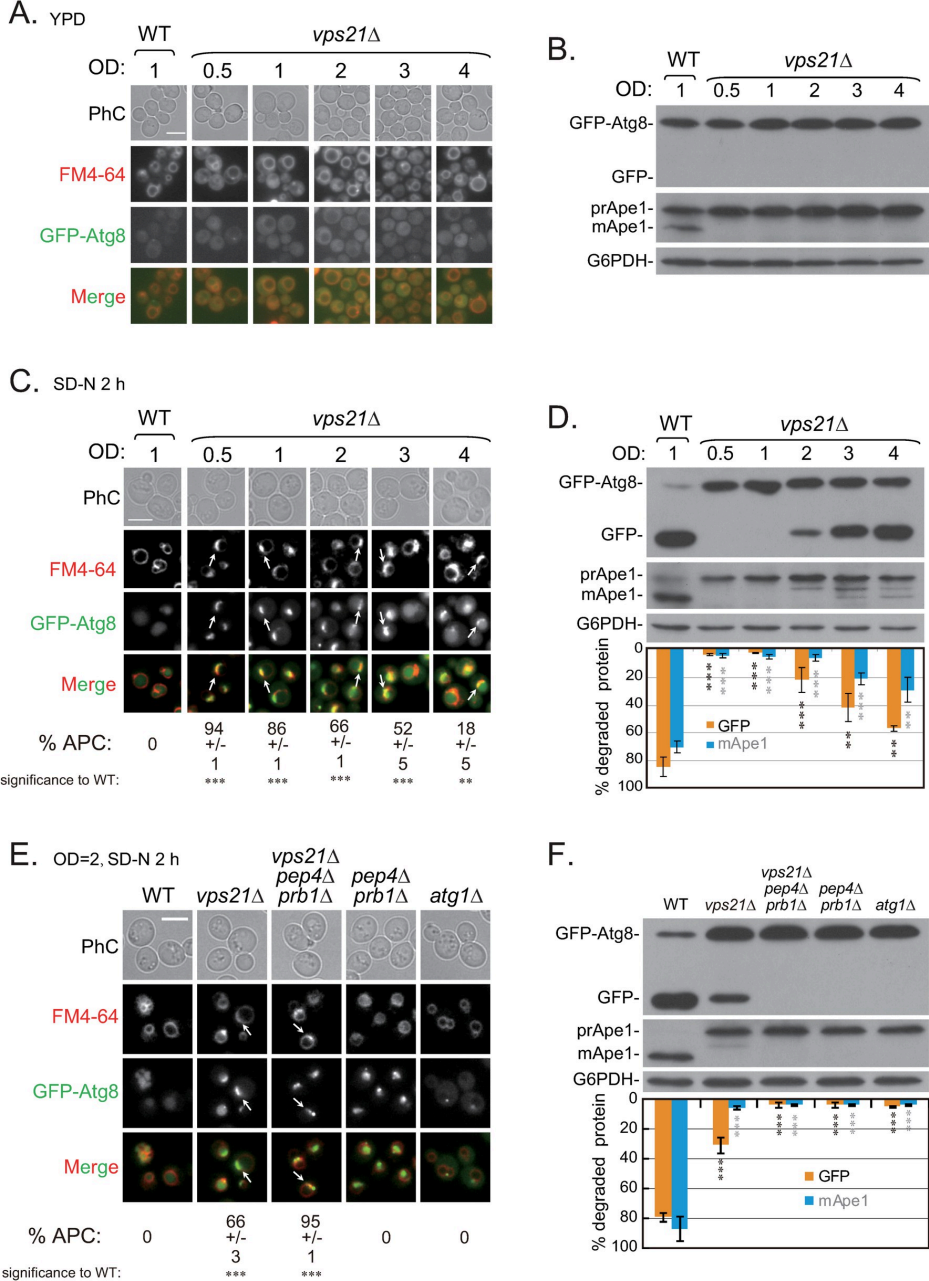
The growth stage before nitrogen starvation and the availability of vacuolar hydrolases impacted the accumulation of autophagosome clusters (APCs) in *vps21Δ* cells. **A.** The increased cell density (OD_600_) of *vps21Δ* cells grown in rich medium did not result in GFP-Atg8 entering into vacuoles. GFP-Atg8-labeled WT and *vps21Δ* cells were grown to the indicated OD_600_ values in rich medium (yeast extract peptone dextrose [YPD] medium) and observed for GFP-Atg8 localization and FM4-64 signals. For WT cells, only the results at an OD_600_ of 1 are presented. **B.** No autophagy processing was detected in *vps21Δ* cells with OD_600_ values ranging from 0.5 to 4. The cells were grown as described in panel A. GFP-Atg8 processing to GFP and prApe1 processing to mApe1 were determined for cell lysates by performing immunoblotting analysis with anti-GFP and anti-Ape1 antibodies, respectively. G6PDH was detected as a loading control. **C.** When starting with a high cell density before nitrogen starvation, the accumulation of GFP-Atg8-labeled APCs in *vps21Δ* cells decreased after nitrogen starvation. WT and *vps21Δ* cells were grown and starved as indicated and stained with FM4-64 for 1 h before being harvested for fluorescence observations. The percentages of cells containing APCs were quantified and are presented below the merged pictures. **D.** When the cell density was high before nitrogen starvation, autophagy processing in *vps21Δ* cells increased after nitrogen starvation. Cells were grown as described in panel C. GFP-Atg8 and prApe1 processing were determined for cell lysates as described in panel B. G6PDH was detected as a loading control. GFP-Atg8 and prApe1 processing were quantified and are presented below the G6PDH blot. **E.** The accumulation of GFP-Atg8-labeled APCs in *vps21Δ* cells increased after nitrogen starvation in the absence of the vacuolar hydrolases Pep4 and Prb1. Cells were grown as described in panel A to an OD_600_ of 2 and starved in SD-N medium for 2 h. The cells were examined and the data are presented as described in panel C. **F.** The partial autophagy processing observed in *vps21Δ* cells after nitrogen starvation was completely blocked in *vps21Δpep4Δprb1Δ* cells. The cells were grown as described in panel E, and autophagy processing was determined and presented as done in panel D. PhC, phase contrast; scale bars in panels A, C, and E, 5 μm; arrows, APCs, and OD, OD_600_. The data shown are presented as the mean +/- the standard deviation (STD). **p < 0.01; ***p < 0.001. Over 600 cells per strain were counted. The results shown represent three independent experiments.

In the first two remarkable yeast autophagy-mutant screenings, relatively few autophagy mutants were screened out [14,15]. Clearly, some autophagy-related proteins were missed from these two screenings, including small GTPases and other proteins [16,17]. Previously, we found that the absence of the Rab5 GTPase Vps21 resulted in the accumulation of phagophore clusters (i.e., unclosed APCs) mostly outside vacuoles after 2 h of nitrogen starvation in SD-N medium [11,18]. However, when cultures with high optical density at 600 nm (OD_600_) values were subjected to nitrogen starvation, the percentages of Vps21-mutant cells with accumulated APCs decreased [11]. These observations suggest that APC accumulation after nitrogen starvation was related to the growth stage. As an autophagosome marker in yeast, the green fluorescent protein (GFP)-Atg8 is delivered to vacuoles through autophagy and degraded to the stable GFP in wild-type (WT) cells. The resulting localization of GFP-Atg8 can be observed by fluorescence microscopy. The yeast precursor form of aminopeptidase I (prApe1) is proteolytically cleaved upon vacuolar delivery or during non-selective autophagy to the mature form of Ape1 (mApe1) in WT cells. The resulting shifts in molecular mass from GFP-Atg8 to GFP or from prApe1 to mApe1 can be monitored by immunoblotting assays [19,20]. To clearly demonstrate the effect of growth-stage on APC accumulation in *vps21Δ* cells, we diluted cells cultured overnight in yeast extract peptone dextrose (YPD) medium to an OD_600_ value of 0.05, after which they were grown to OD_600_ values of 0.5, 1, 2, 3, or 4. The cells were stained with FM4-64 dye for 1 h before they were collected for fluorescence microscopy observations. Comparable localizations of GFP-Atg8 to the cytosol but not to FM4-64-labelled vacuoles were observed in WT cell (OD_600_ = 1) and *vps21Δ* cells (at different OD_600_ values) (Fig 1A). Immunoblotting analysis showed that GFP-Atg8 was not degraded in WT or *vps21Δ* cells under any conditions investigated (Fig 1B). In contrast, prApe1 did not mature to mApe1 in *vps21Δ* cells due to defective vacuolar delivery under normal growth conditions, whereas prApe1 partially matured in WT cells (OD_600_ = 1) (Fig 1B). Because GFP-Atg8 entry into vacuoles in WT cells was not affected by varying the cell density (OD_600_ = 0.5–4, before nitrogen starvation) (Fig 1A and 1B), we only presented data for WT cells grown to OD_600_ = 1 from here onward. Interestingly, when *vps21Δ* cells grown to various cell densities (OD_600_ = 0.5–4) were adjusted to a similar cell density (OD_600_ = ~0.8–1) and subjected to nitrogen starvation for 2 h, the cells starting from the lowest cell density (OD_600_ = 0.5) showed the highest percentage (94%) of cells with APCs around the vacuole membranes, whereas those starting from the highest cell density (OD_600_ = 4) showed the lowest percentage (~20%) (Fig 1C). Moreover, the number of cells with GFP-positive vacuoles increased as the cell density increased (Fig 1C). GFP-Atg8 degradation and prApe1 maturation in *vps21Δ* cells increased as the cell density increased, suggesting that autophagy became elevated (Fig 1D). These data indicate that, although the late growth stage itself before nitrogen starvation did not result in increased autophagy, *vps21Δ* cells at a late growth stage are able to accumulate fewer APCs after a short period of nitrogen starvation.

Previous reports showed that the levels of vacuolar proteases changed in a growth-stage-dependent manner and peaked when the cells approached the stationary phase [12,13]. Therefore, the decreased APC accumulation in *vps21Δ* cells starting from the high cell density might have been partially caused by increased vacuolar protease levels. Conversely, a decreased level or absence of vacuolar proteases might have led to increased APC accumulation. Indeed, we noticed that the percentages of Vps21- or ESCRT-mutant cells displaying accumulated APCs was higher in the absence of the Pep4 hydrolase [10,11,18].

To confirm these observations, we constructed WT and *vps21Δ* strains that lacked two key vacuolar proteases (Pep4 and Prb1). Then, we examined the status of GFP-Atg8 localization and autophagy processes after the cells were intentionally cultured to a relatively high cell density (OD_600_ = 2) and subjected to nitrogen starvation for 2 h. The percentage of cells displaying APCs increased from 66% in *vps21Δ* cells to 95% in *vps21Δpep4Δprb1Δ* cells under the same growth conditions (Fig 1E). As expected, autophagy was completely blocked in *vps21Δpep4Δprb1Δ* cells, similar to the results found with *pep4Δprb1Δ* and *atg1Δ* cells (Fig 1F). These results confirmed the roles of vacuolar hydrolases in APC accumulation in *vps21Δ* cells after a short period of nitrogen starvation. Collectively, these findings indicate that unclosed APC accumulation in *vps21Δ* cells depended on the growth stage and expression levels of hydrolases. A high percentage of *vps21Δ* cells lacking hydrolases could still accumulate APCs at the late growth stage. These data suggest that some APCs in *vps21Δ* cells might have entered vacuoles when the cells were during the late growth stage.

### Unclosed APCs in *vps21Δ* cells lacking key vacuolar proteases enter vacuoles after a prolonged period of autophagy induction

**Fig 2:**
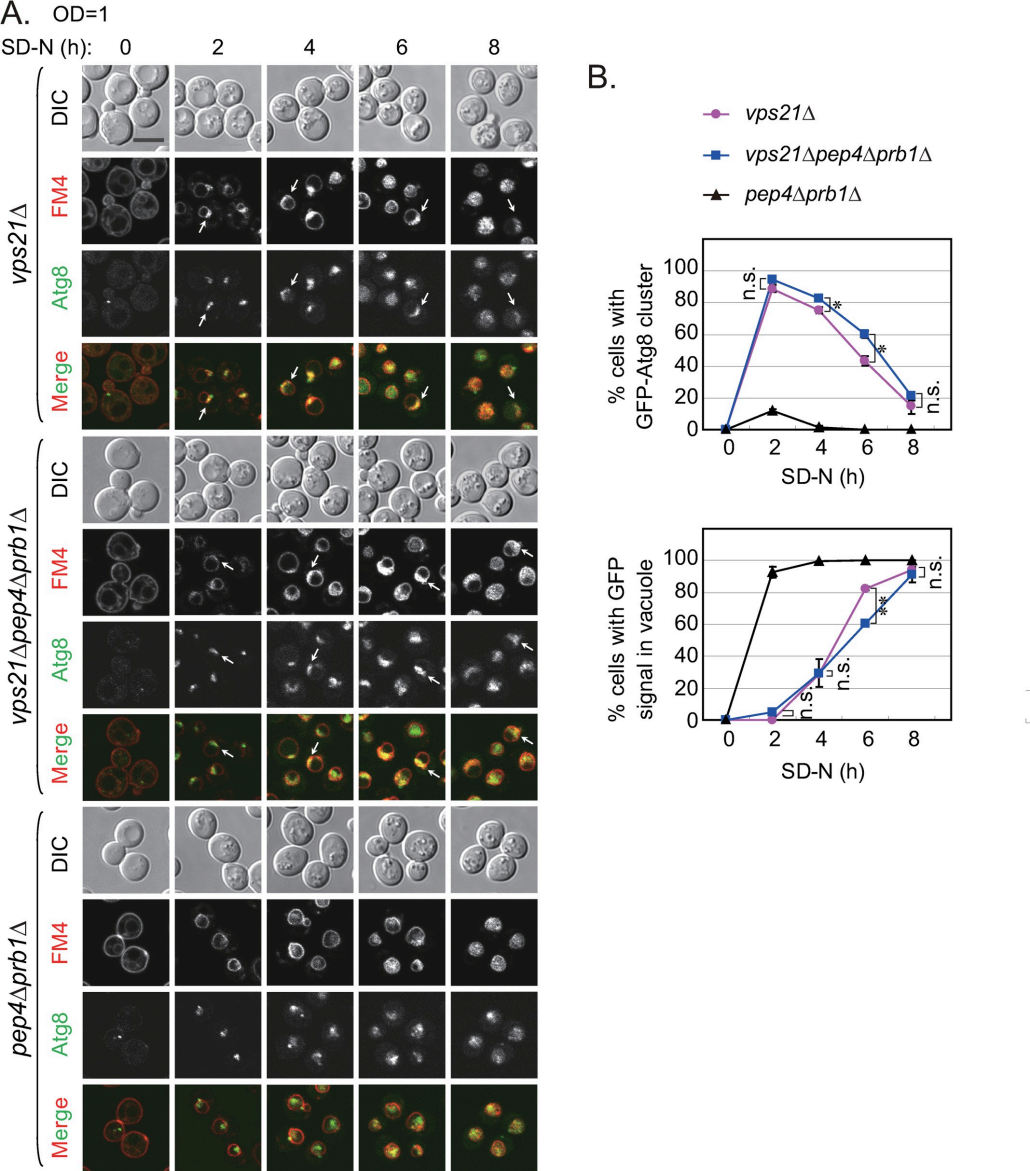
The accumulated APCs in *vps21Δ* cells entered vacuoles after prolonged nitrogen starvation. **A.** The accumulated GFP-Atg8-labeled APCs observed in *vps21Δ* cells gradually entered vacuoles during prolonged nitrogen starvation. The indicated cells expressing GFP-Atg8 (as in Fig 1E) were grown in YPD medium as described in Fig 1A to an OD_600_ value of 1 and then starved in SD-N medium for the indicated durations before fluorescence observations were made. FM4-64 was added 1 h before the cells were collected for visualization by fluorescence microscopy. DIC, differential- interference contrast; scale bar, 5 μm; arrows, APCs. **B.** Quantification of the cells containing APCs and GFP signals in vacuoles shown in panel A. Under prolonged nitrogen starvation, the percentage of *vps21Δ* and *vps21Δpep4Δprb1Δ* cells (top) containing APCs peaked after 2 h of nitrogen starvation and subsequently declined, whereas the percentage of *vps21Δ* and *vps21Δpep4Δprb1Δ* cells containing GFP signals in vacuoles gradually increased in the cells (bottom). However, GFP-Atg8 increasingly entered vacuoles in *pep4Δprb1Δ* cells to accumulate under prolonged nitrogen starvation (bottom). The data shown are presented as the mean +/- STD. *p < 0.05; **p < 0.01; n.s., not significant. Over 500 cells per strain were counted. The results shown represent two independent experiments.

In the absence of vacuolar proteases, if APCs were to enter vacuoles during autophagy progression, then these non-degraded APCs should be easily observed. To determine whether unclosed APCs in *vps21Δ* cells could enter vacuoles when the key vacuolar proteases were absent, we grew *vps21Δ*, *vps21Δpep4Δprb1Δ*, and *pep4Δprb1Δ* cells that expressed GFP-Atg8 in YPD medium until the OD_600_ reached 1. Then, we starved the cells in SD-N medium for different times (0–8 h) to observe the localizations of GFP-Atg8-labeled APCs. The percentage of *vps21Δ* cells displaying APCs peaked at 2 h after nitrogen starvation and gradually decreased afterwards, whereas that displaying GFP-positive vacuoles increased as the starvation progressed (Fig 2A and 2B). Similar results were found with *vps21Δpep4Δprb1Δ* cells. However, significantly more APC accumulation and less vacuolar GFP signals were observed in *vps21Δpep4Δprb1Δ* cells at 6 h of nitrogen starvation (Fig 2A and 2B). In *pep4Δprb1Δ* cells, closed APs entered vacuoles to form ABs but were not degraded given the absence of vacuolar hydrolases. The number of ABs increased gradually as the starvation progressed (Fig 2A and 2B). These results indicate that the unclosed APCs that accumulated around vacuole membranes in *vps21Δ* and *vps21Δpep4Δprb1Δ* cells might have entered vacuoles after prolonged nitrogen starvation.

In addition to nitrogen starvation, rapamycin treatment is another common inducer of autophagy that is used in laboratory settings for both yeast and mammalian cells [21,22]. Like nitrogen starvation, rapamycin treatment induced APC accumulation in *vps21Δ* cells [11,23]. As controls, we grew cells in SD-N or YPD medium without rapamycin to test whether the APCs induced by rapamycin treatment in *vps21Δ* cells also enter vacuoles after a prolonged induction period. Because FM4-64 staining significantly delayed rapamycin-induced APC accumulation and APC entry into vacuoles, the FM4-64-staining step was omitted in this experiment. The percentage of *vps21Δ* cells with APCs gradually increased following rapamycin treatment, peaked at approximately 6 h, and declined afterwards, along with increased GFP signals in vacuoles. These results were similar to those with nitrogen starvation, except that the APCs showed earlier and increased accumulation and entered the vacuoles faster with nitrogen starvation (S1A and S1B Fig). As expected, control *vps21Δ* cells that were not treated with rapamycin during the same incubation period did not show induced APC accumulation (S1A Fig). Immunoblotting assays showed that GFP-Atg8 and prApe1 processing in *vps21Δ* cells after rapamycin treatment were similar to those after nitrogen starvation but occurred approximately 4 h later (S1C Fig). These results indicate that both nitrogen starvation and rapamycin treatment induced the entry of unclosed APCs into vacuoles in *vps21Δ* cells.

**Fig 3:**
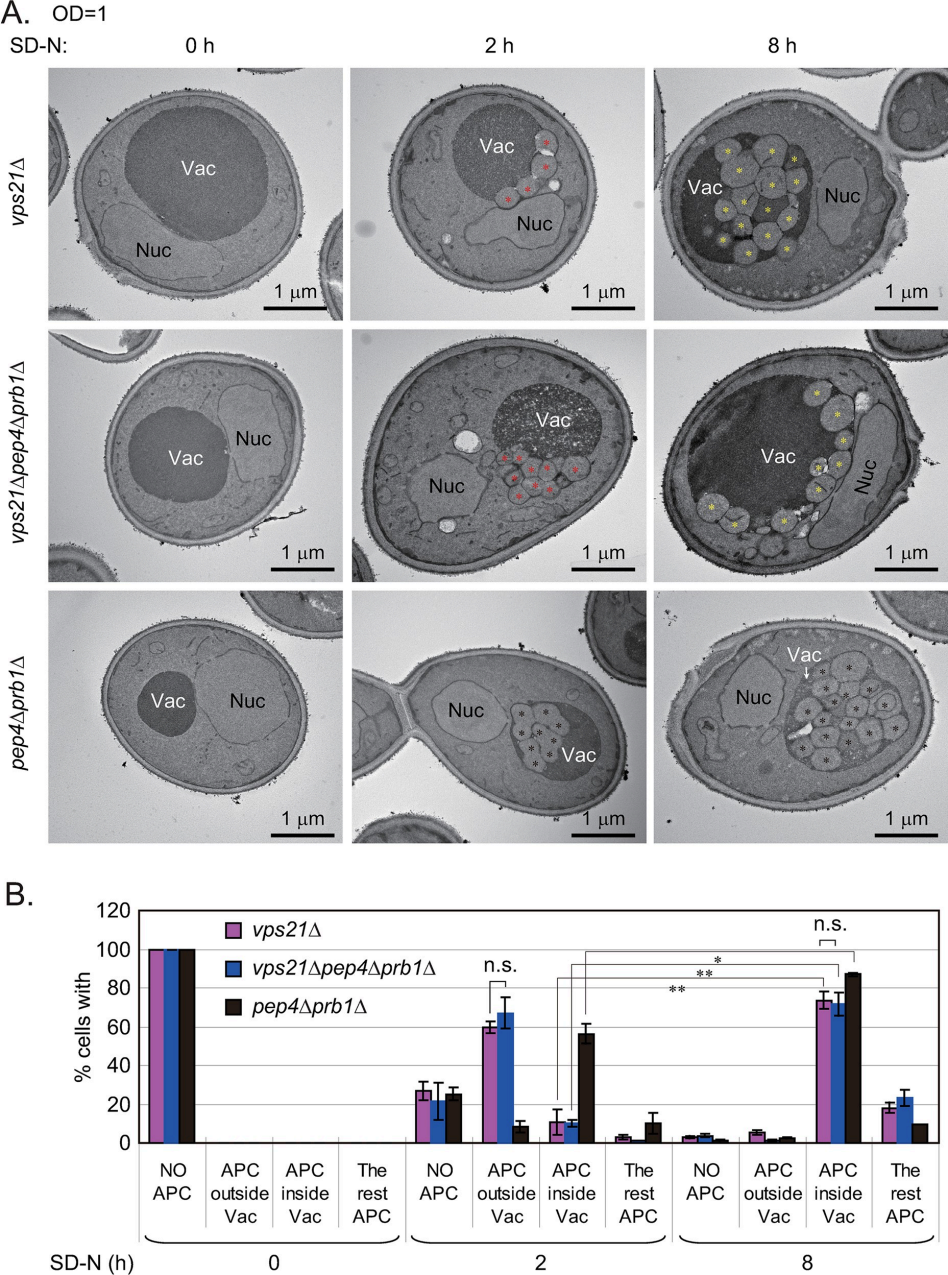
Ultrastructural analysis of APCs in *vps21Δ* cells after representative durations of nitrogen starvation. Cells were grown and treated as described in Fig 2A but were not stained with FM4-64. **A.** Cells after 0, 2 or 8 h of nitrogen starvation were fixed and subjected to transmission electron microscopy (TEM) analysis as described [11]. We referred to the autophagosome-like structures outside vacuoles as autophagosomes (APs) and to those inside vacuoles as autophagic bodies (ABs). If they were found in clusters, they were referred as AP clusters (APCs) or AB clusters (ABCs). Vac, vacuole; Nuc, nucleus; red asterisks, APs; yellow asterisks, ABs; black asterisks, closed ABs. **B.** Quantification for cells containing APCs in panel A. Cells were divided into four categories regarding APCs: NO APCs, no obvious APCs; APC outside Vac, greater than or equal to half of the AP-like structures resided outside of vacuoles; APCs inside Vac, over half of the AP-like structures (APs and/or ABs) resided inside vacuoles; the rest, difficult to distinguish whether the AP-like structures resided inside or outside of vacuoles. The columns represent the mean and the error bars represent the STD. *p < 0.05; **p < 0.01; n.s., not significant. Over 400 slices were counted for each strain following each treatment. The results shown represent two independent experiments.

We wanted to confirm the entry of APCs into vacuoles via ultrastructural analysis. Thus, we performed TEM to examine the cells represented in Fig 2A (0, 2, and 8 h). We divided the cells with accumulated APCs (relative to vacuole membranes) into four categories, quantified the percentages in each category, and confirmed that the percentages of cells displaying APCs inside the vacuoles increased significantly after 8 h of nitrogen starvation (Fig 3A and 3B). No APCs were found in any cells before nitrogen starvation, but APCs were present in *vps21Δ* and *vps21Δpep4Δprb1Δ* cells after 2 h of nitrogen starvation and were mainly localized outside of the vacuoles (Fig 3A and 3B). Moreover, APCs (or more accurately, AB clusters [ABCs]) were detected inside the vacuoles of *pep4Δprb1Δ* cells after 2 h of nitrogen starvation (Fig 3A and 3B). After 8 h of nitrogen starvation, the percentages of *vps21Δ* and *vps21Δpep4Δprb1Δ* cells with APCs inside their vacuoles had increased significantly, and the percentage of *pep4Δprb1Δ* cells with APCs/ABCs inside their vacuoles had also increased significantly (Fig 3A and 3B).

**Fig 4:**
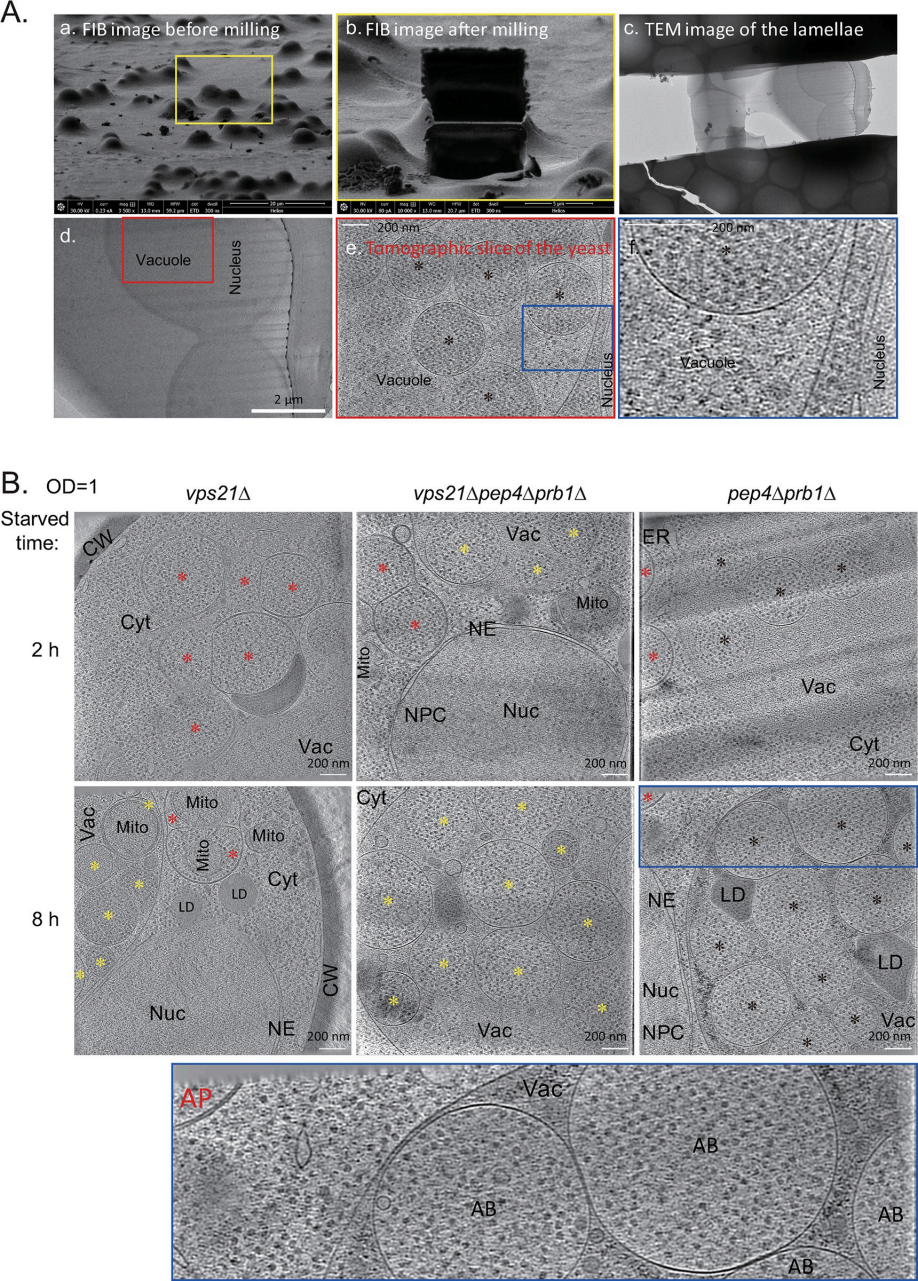
Cryo-focus ion beam (Cryo-FIB) and cryo-electron tomography (Cryo-ET) analyses of APCs in *vps21Δ* cells under conditions representative of nitrogen starvation. Cells of *vps21Δ* were grown and treated as described in Fig 3A. After subjecting the cells to nitrogen starvation for 2 or 8 h, they were immediately analyzed using the Cryo-FIB and Cryo-ET methods, as described in the Materials and methods section. **A.** The major steps of Cryo-FIB and Cryo-ET are shown from the left top to the right bottom: a. FIB image before milling; b. FIB image after milling; c. TEM image of the lamellae; d. magnified TEM image; e. tomographic slice of the yeast cells; f. magnified tomographic slice of the yeast cells. The same color frame indicates the same area. **B.** Tomographic slice of the nucleus–vacuole junction area of the indicated yeast cells starved for 2 or 8 h. The blue framed area of *pep4Δprb1Δ* cells after 8 h of nitrogen starvation was magnified below to show the image in greater details. Red asterisks, APs with a double-membrane; yellow asterisks, ABs with a single-membrane; black asterisks, closed ABs with a single-membrane; CW, cell wall; Cyt, cytoplasm; Vac, vacuole; Nuc, nucleus; NE, nuclear envelope; NPC, nuclear pore complex; Mito, mitochondria; ER, endoplasmic reticulum; LD, lipid droplet. See the supplemental video (S1–S6 Movies) for tomograms.

Previously, we showed that membranes of APCs facing the cytosol in Vps21-module mutants are double-membraned [18]. However, regular TEM could not distinguish whether the APCs inside the vacuoles of *vps21Δ* and *vps21Δpep4Δprb1Δ* cells were double-membraned or single-membraned (Fig 3). To overcome this technical difficulty, Cryo-FIB and Cryo-ET analyses [24] were performed with APCs in cells after 2 or 8 h of nitrogen starvation. As shown in Fig 4A, the double-membrane structure of the nucleus and the single-membrane structure of vacuoles were distinguishable by Cryo-FIB and Cryo-ET. After 2 h of nitrogen starvation, the double-membrane AP structures outside vacuoles and the single-membrane AP-like structures inside vacuoles displayed similar electron-density profiles of the cytosol, which differed from those in vacuolar lumens with all cell types studied (Fig 4B and S1–S3 Movies). When the cells were starved for 8 h, more single-membrane AP-like structures accumulated inside the vacuoles in all cell types studied, whereas double-membrane AP structures outside vacuoles were seen occasionally (Fig 4B and S4–S6 Movies). We refer to these single-membrane AP-like structures inside vacuoles here as ABs ([25] and Fig 4B).

**Fig 5:**
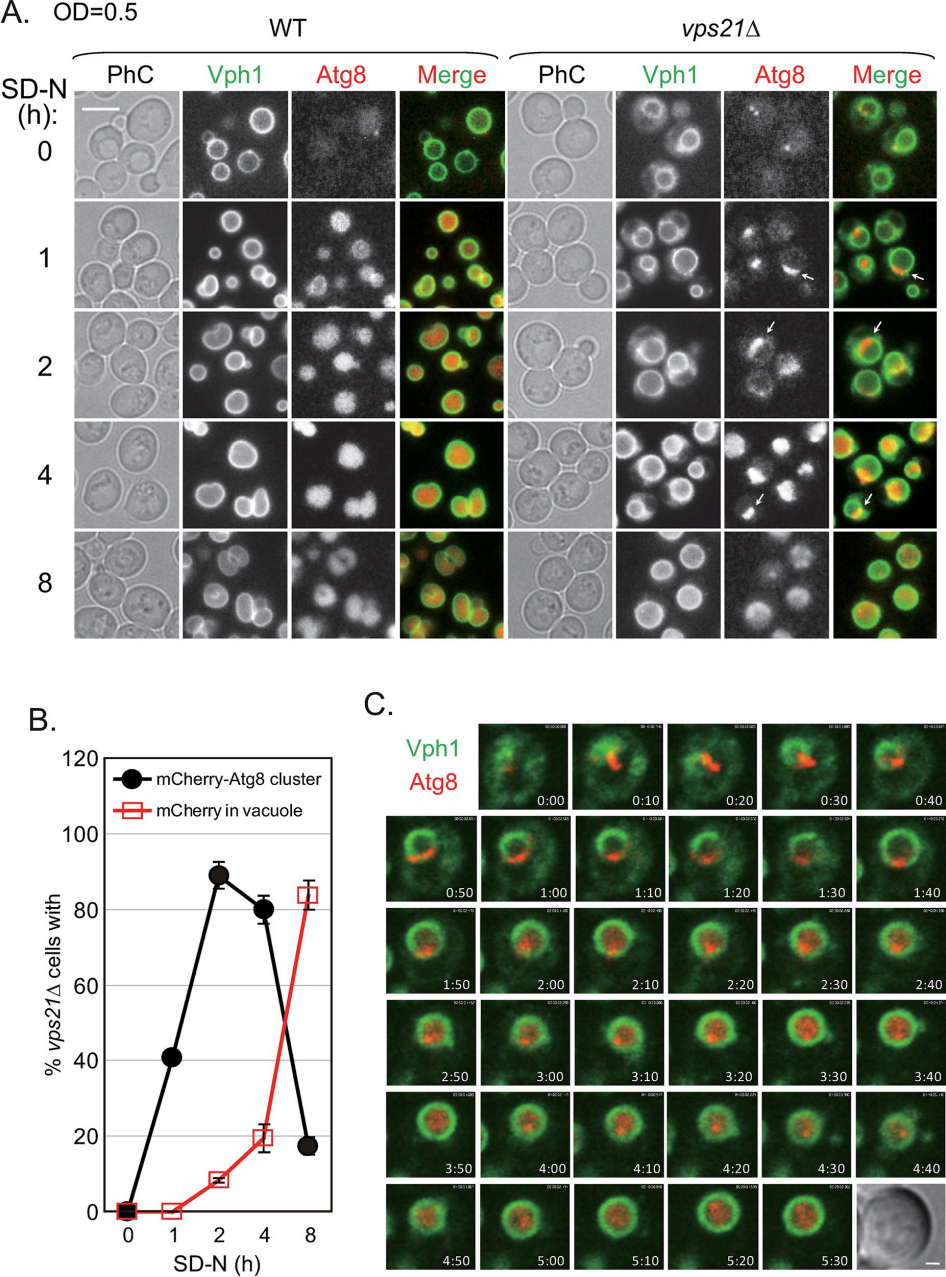
The entry of accumulated mCherry-Atg8-labeled clusters into *vps21Δ* cell vacuoles after prolonged nitrogen starvation. WT and *vps21Δ* cells labeled with Vph1-GFP and mCherry-Atg8 were subjected to prolonged nitrogen starvation as described in Fig 2A, except that the cell density was set to an OD_600_ of 0.5 before nitrogen starvation in order to show more Atg8 clusters. **A.** The accumulated mCherry-Atg8 clusters on Vph1-GFP-labeled vacuole membranes in *vps21Δ* cells entered the vacuoles after prolonged nitrogen starvation. In contrast, the mCherry-Atg8 signal entered vacuoles in WT cells quickly after nitrogen starvation. Scale bar, 5 μm. The arrows indicate mCherry-Atg8 clusters on vacuole membranes. **B.** Quantification of *vps21Δ* cells containing mCherry-Atg8 clusters and mCherry in vacuoles. The data shown are presented as the mean +/- STD. **C.** Time-lapse fluorescence microscopy showed that the mCherry-Atg8 clusters of *vps21Δ* cells entered vacuoles after prolonged nitrogen starvation. The *vps21Δ* cells were grown and starved as described in Fig 5A for 1 h and loaded onto a solid SD-N-medium pad on a glass slide for time-lapse fluorescence microscopy observations with 1 min intervals. Sequential overlapping frames showing mCherry-Atg8 and Vph1-GFP fluorescence at 10 min intervals are shown in the still images, with DIC at time zero. Scale bar, 1 μm. See the supplemental video of mCherry-Atg8 and Vph1-GFP in *vps21Δ* cells with 1 min intervals at 5.5 h and a play rate of 10 frames/s (fps) (S7 Movie) for further details. The results shown represent at least two independent experiments.

The data above indicate that the AP structures located outside of the vacuoles of *vps21Δ* and *vps21Δpep4Δprb1Δ* cells might have robustly entered vacuoles during the period between 2 and 8 h of nitrogen starvation (Figs 2 and 4). FM4-64 stained both APCs and vacuoles in *vps21Δ* cells during nitrogen starvation ([18] and Figs 1 and 2), which made it difficult to distinguish whether APCs were localized inside or outside vacuoles based on the GFP-Atg8 and FM4-64 signals. To visualize the entry of APCs into vacuoles during nitrogen starvation, we labeled the vacuole membranes and APs with Vph1-GFP and mCherry-Atg8, respectively (Fig 5A). We first observed the fluorescence in WT and *vps21Δ* cells expressing Vph1-GFP and mCherry-Atg8 at various time points after nitrogen starvation (Fig 5A). The percentage of *vps21Δ* cells with mCherry-Atg8-labeled APCs peaked after 2 h of nitrogen starvation and gradually decreased afterwards, and the corresponding mCherry signal in the vacuoles increased gradually (Fig 5A and 5B). These data are consistent with the results shown in Fig 2. Next, we performed live-cell confocal microscopy to visualize APCs entering vacuoles in *vps21Δ* cells. Briefly, the cells were starved in liquid SD-N medium for 1 h and observed on a solid SD-N-medium pad on a glass slide. Our results clearly showed that APCs located outside of the vacuoles or on the vacuole membranes gradually entered the vacuoles (Fig 5C and S7 Movie). Together, these results provided **direct** evidence that unclosed APs can enter vacuoles after prolonged nitrogen starvation.

**Fig 6:**
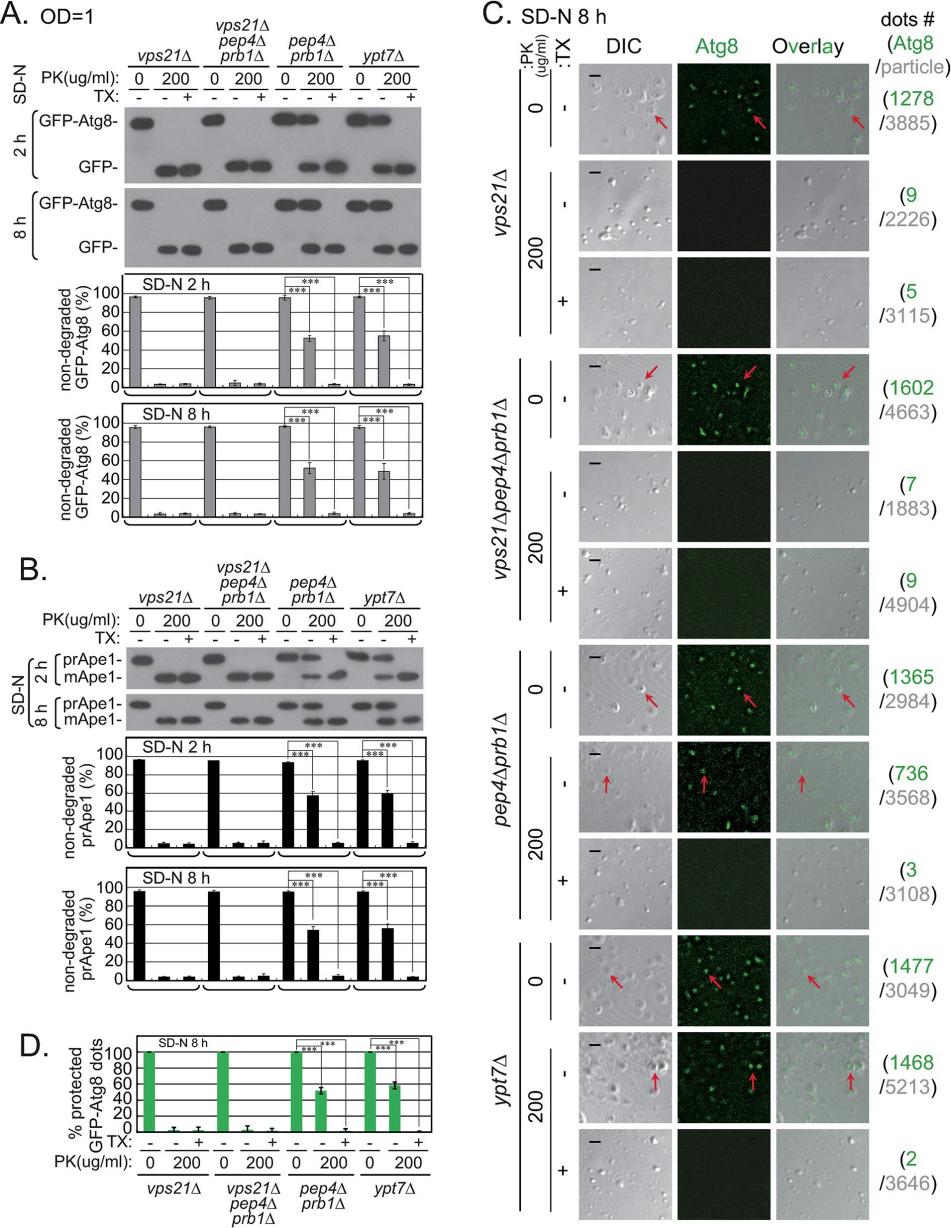
AP-related membrane structures that entered vacuoles of *vps21Δ* cells after prolonged nitrogen starvation were sensitive to protease K (PK) digestion. Cells were grown as described in Fig 2A and subjected to a conventional protease K (PK)-protection assay as described [10,45] or a modified microscopy-based PK-protection assay [10,26]. The *ypt7Δ* and *pep4Δprb1Δ* cells served as controls for closed APs outside vacuoles and closed ABs inside vacuoles, respectively. **A-B.** Performing the conventional PK-protection assay combined with immunoblotting analysis showed that GFP-Atg8 (A) or prApe1 (B) on AP-related membrane structures (pellet) isolated from *vps21Δ* and *vps21Δpep4Δprb1Δ* cells grown under prolonged nitrogen starvation (8 h) were still sensitive to PK. The top blots in panels A-B represent pellets from cells starved in SD-N medium for 2 h, and the bottom blots represent pellets from cells starved for 8 h. Protease protection of GFP-Atg8 or prApe1 was detected when PK was added to the membranes without detergent. The levels of non-degraded GFP-Atg8 and prApe1 were quantified and are presented below the blots. **C.** The modified microscopy-based PK-protection assay showed that GFP-Atg8 in AP-related membrane structures isolated from *vps21Δ* and *vps21Δpep4Δprb1Δ* cells grown under prolonged nitrogen starvation (8 h) were accessible to PK. Following the steps of the conventional PK-protection assay, but the fractions were observed by fluorescence microscopy instead of immunoblotting analysis. GFP-Atg8 was not observed in particles from *vps21Δ* and *vps21Δpep4Δprb1Δ* cells when PK was added to membranes without detergent. The results for the AP-related membrane structures isolated from cells grown in SD-N medium for 8 h are presented here. The data obtained after 2 h in SD-N medium are presented in S1D Fig. Scale bars, 2 μm; arrows point to representative GFP-Atg8-positive particles. The right column indicates the total GFP-Atg8-positive dots in green numbers and particles in grey numbers from three independent experiments used for quantification in panel D. **D.** Quantification of the percentages of GFP-Atg8 particles protected from PK for the strains shown in panel C; >1800 DIC particles were quantified for each condition (1–2 fields × 3 replicates). The columns in panels A-B and D represent the mean, and the error bars represent the STD. ***p < 0.001.

It has been proposed that upon entering vacuoles, closed double-membrane APs become single-membrane ABs [6], as demonstrated in Fig 4. Interestingly, the unclosed double-membrane APs outside the vacuoles in *vps21Δ* and *vps21Δpep4Δprb1Δ* cells changed to single-membrane ABs within vacuoles, with a similar morphology to the closed single-membrane ABs within vacuoles in *pep4Δprb1Δ* cells (Fig 4). Thus, we performed a conventional protease K-protection (PK) assay as reported [11] to determine whether the single-membrane ABs within vacuoles in *vps21Δ* and *vps21Δpep4Δprb1Δ* cells were closed. Consistent with previous findings [10,11], the AP-related membrane structures in *pep4Δprb1Δ* or *ypt7Δ* cells were closed after 2 h of nitrogen starvation, and the AP-related membrane structures residing outside of the vacuoles in *vps21Δ* and *vps21Δpep4Δprb1Δ* cells were unclosed (Fig 6A and 6B). The characteristic membrane features of AP-related membrane structures were the same even after 8 h of nitrogen starvation (Fig 6A and 6B).

We used a modified microscopy-based PK assay, developed in our laboratory previously [10,26], to test these AP-related membrane structures further. Regardless of whether APs/ABs were inside the vacuoles of *pep4Δprb1Δ* cells or outside the vacuoles of *ypt7Δ* cells, they were isolated as markedly smaller particles (approximately 500 nm in diameter) than regular vacuoles (approximately 1–2 μm in diameter) by microscopy observations. These observations indicate that the APs/ABs residing inside of vacuoles exited the vacuoles or at least these APs/ABs were not isolated along with intact vacuoles. Particles isolated from APs/ABs residing inside or outside of vacuoles of *vps21Δ* or *vps21Δpep4Δprb1Δ* cells grown in SD-N medium for 2 or 8 h were not protected against protease digestion, whereas particles isolated from *pep4Δprb1Δ* or *ypt7Δ* cells under the same growth and treatment conditions were partially protease-protected. Because when protease K (but not detergent) was added to the isolated particles, GFP signals completely disappeared in particles from *vps21Δ* and *vps21Δpep4Δprb1Δ* cells but only partially decreased in particles from *pep4Δprb1Δ* or *ypt7Δ* cells. When protease K and detergent were added together to the isolated particles, GFP signals completely disappeared in particles from all cell types (Figs 6C, 6D and S1D). These results indicate that the AP-related membrane structures in *vps21Δ* and *vps21Δpep4Δprb1Δ* cells were unclosed regardless of whether they resided inside or outside of vacuoles (Fig 6). Therefore, when *vps21Δ* or *vps21Δpep4Δprb1Δ* cells were grown in SD-N medium for 8 h, we refer to their APs as unclosed ABs when they are inside vacuoles and are single-membraned (Fig 4).

The dissociation of several Atgs from APs is a hallmark of AP maturation, whereas Atg8 remains on APs until they fuse with vacuoles [27]. Previous findings showed that unclosed APs as well as the closed (but not completely matured) APs were associated with Atg proteins, in contrast to Atgs that dissociated from mature APs in cytosol [10,11,28]. Here, we found that the unclosed double-membrane APCs that localized outside vacuoles in *vps21Δ* cells after 2 h of nitrogen starvation entered vacuoles to become unclosed single-membrane ABs after 8 h of nitrogen starvation (Figs 2 and 6). We hypothesized that the Atgs that decorated the unclosed double-membrane APCs outside of vacuoles might be released, such that they were not present on unclosed single-membrane ABs inside of vacuoles. To test this hypothesis, we compared the colocalization of representative marker Atg11-GFP with mCherry-Atg8 in *vps21Δ* and *vps21Δpep4Δ* cells after nitrogen starvation for 2 or 8 h. We observed clear colocalization between Atg11 and Atg8 that accumulated in crescent form around the vacuole membranes in *vps21Δ* and *vps21Δpep4Δ* cells after nitrogen starvation for 2 h. However, only Atg8 showed a crescent-like distribution within vacuoles after nitrogen starvation for 8 h. After the cells were under nitrogen starvation for 2 or 8 h, most Atg11 was released from Atg8 dots/puncta in (i) *ypt7Δ* cells containing closed APs in the cytosol or (ii) *pep4Δ* cells containing closed ABs within vacuoles (S2 Fig). These results further confirmed that the accumulated unclosed APCs outside vacuoles entered vacuoles and formed unclosed ABs in *vps21Δ* cells.

### Proteins are involved in the entry of unclosed APCs into vacuoles in *vps21Δ* cells during nitrogen starvation

**Fig 7:**
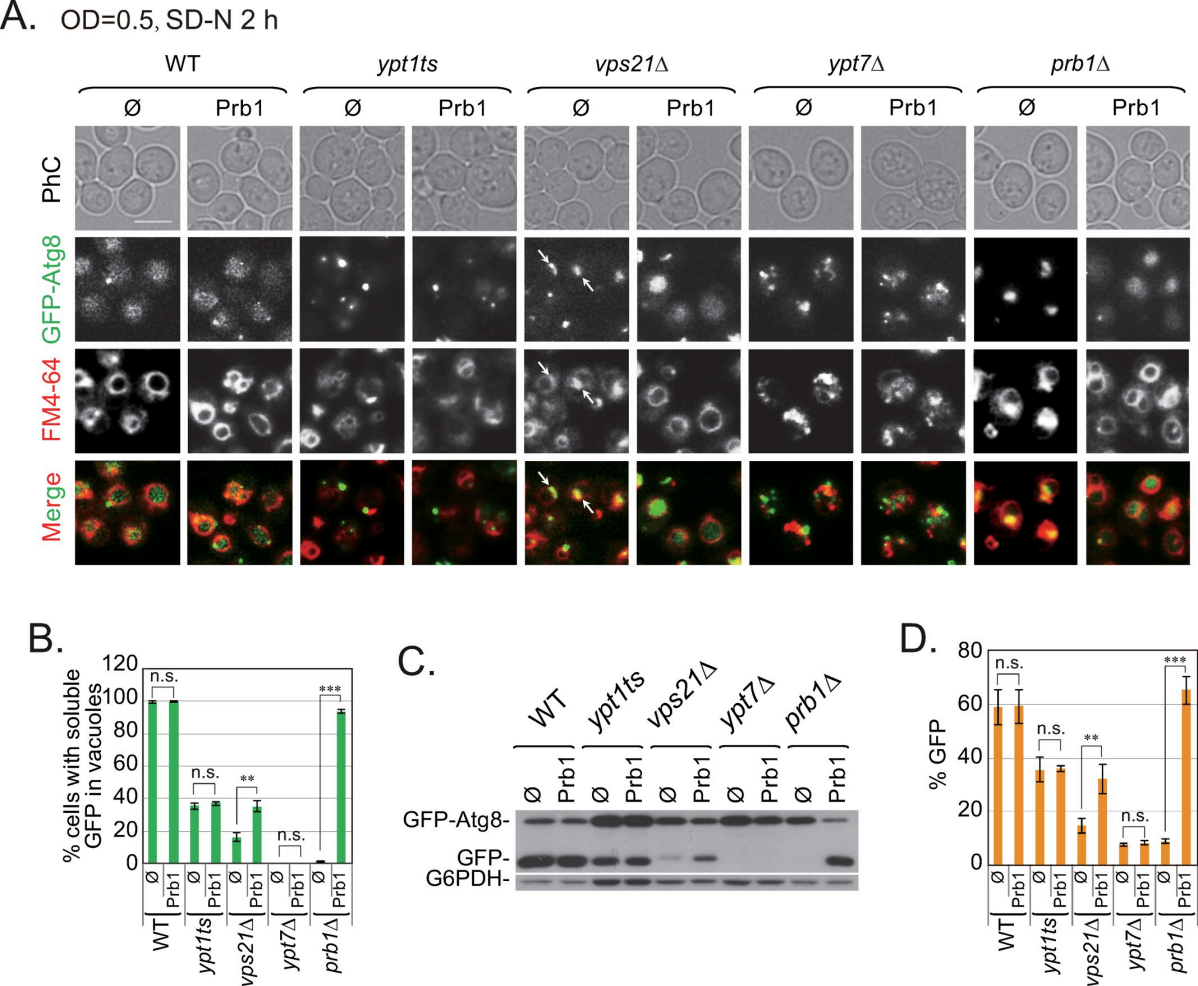
Prb1 overexpression promoted APC entry into vacuoles in *vps21Δ* cells (but not in *ypt1ts* and *ypt7Δ* cells) after nitrogen starvation. **A.** The accumulation of GFP-Atg8-labeled APCs in *vps21Δ* cells decreased after Prb1 overexpression. The indicated cells were transformed with a Prb1-expression plasmid or the empty vector (pRS415, ∅), grown to log phase, and starved in SD-N for 2 h with FM4-64 staining for 1 h before being harvested for fluorescence observations. Scale bar, 5 μm; arrows, APCs. **B.** Quantification of cells containing soluble GFP in their vacuoles. The percentages of cells containing diffused GFP in vacuoles in panel A were quantified and are presented as the mean +/- STD. Over 600 cells were counted for each strain. **C.** GFP-Atg8 degradation in *vps21Δ* cells increased after Prb1 overexpression. The cells were grown as described in panel A and examined for GFP-Atg8 degradation as described in Fig 1. G6PDH was detected as a loading control. **D.** Quantification of GFP-Atg8 degradation represented in the immunoblot shown in panel C. The quantification was performed as described in Fig 1D for GFP-Atg8 degradation and the data are presented as the mean +/- STD. P values in B and D: n.s., not significant; **p < 0.01; ***p < 0.001. The results shown represent at least two independent experiments.

If the reduced accumulation of APCs in *vps21Δ* cells was due to increased levels of vacuolar hydrolases when the cells were cultured to a high cell density before nitrogen starvation (Fig 1), then we would expect that overexpressing vacuolar hydrolases would facilitate the entry of APCs into vacuoles. To test this hypothesis, we overexpressed a functional hydrolase (Prb1) in *vps21Δ* cells and found that fewer cells displayed APCs (Fig 7A). Consistently, the percentage of *vps21Δ* cells displaying soluble GFP signals inside their vacuoles increased upon Prb1 overexpression (Fig 7A and 7B). As Vps21 is a Rab GTPase, we also tested whether the Prb1 overexpression could affect the GFP-Atg8 phenotypes in Rab GTPase-mutant *ypt1ts* and *ypt7Δ* cells. We found that Prb1 overexpression did not affect the GFP-Atg8 phenotypes in *ypt1ts* and *ypt7Δ* cells (Fig 7A and 7B). Consistently, Prb1 overexpression only promoted GFP-Atg8 degradation in *vps21Δ* cells, but not in *ypt1ts* and *ypt7Δ* cells (Fig 7C and 7D). Overexpressing another vacuolar hydrolase (Pep4) in *vps21Δ* cells had similar effects (S9A and S9B Fig). These results validated our hypothesis.

We further searched the literature for other proteins that might promote the entry of unclosed APCs into vacuoles in *vps21Δ* cells. VTC-complex proteins have been implicated in several aspects of membrane transport and vesicular traffic, including microautophagy [29]. Recently, VTC proteins were reported to negatively regulate the vacuolar uptake of misfolded glycosylphosphatidylinositol-anchored proteins in *pep4Δ* cells [30]. We examined whether VTC proteins were required for the entry of unclosed APCs into *vps21Δ* cell vacuoles. We deleted *VTC1-5* individually from GFP-Atg8-labeled WT and *vps21Δ* cells and monitored GFP-Atg8 entry into FM4-64-stained vacuoles after nitrogen starvation for 0, 2 or 8 h. Depleting Vtc1-5 individually from WT cells did not clearly affect GFP-Atg8 entry into vacuoles with increasing nitrogen-starvation times. However, when Vtc1, 2, 4, or 5 was depleted individually from *vps21Δ* cells, the APCs that accumulated outside vacuoles in *vps21Δ* cells after 2 h of nitrogen starvation were subsequently delivered into vacuoles. GFP-Atg8 in the vacuoles of these double mutants were detected in particulates but were dispersed better than those in *pep4Δ* cells. No clear difference was found in terms of GFP-Atg8 entry into vacuoles between *vps21Δ* and *vps21Δvtc3Δ* cells (S3A Fig).

**Fig 8:**
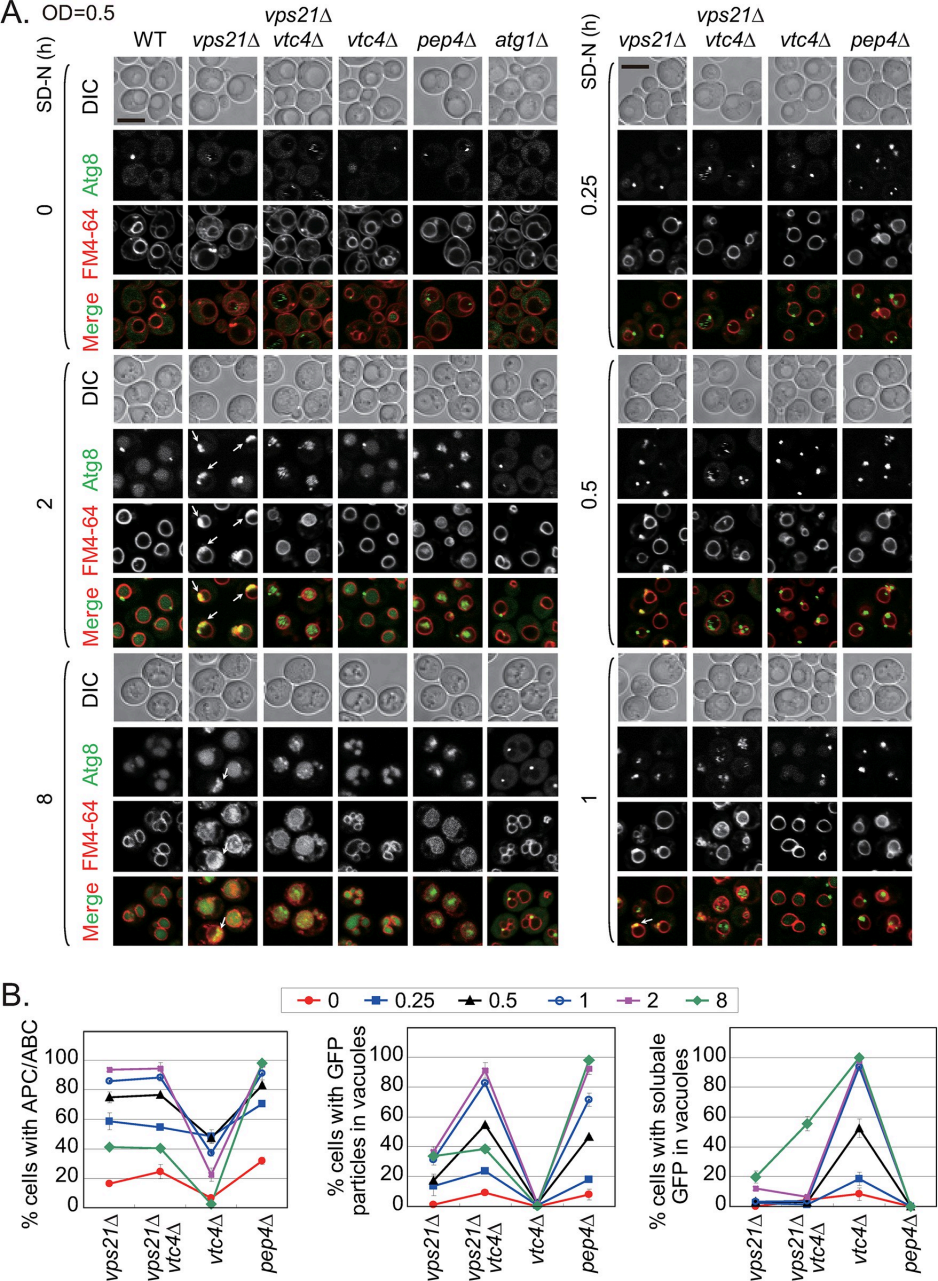
Vtc4 inhibited APC entry into vacuoles in *vps21Δ* cells under nitrogen starvation. **A.** Vtc4 depletion promoted the entry of accumulated APCs into vacuoles in *vps21Δ* cells under nitrogen starvation. The indicated GFP-Atg8-labeled cells were grown and examined as described in Fig 2. The four core strains incubated in SD-N medium between 0–2 h are presented on the right side. Scales bars, 5 μm; arrows, APCs. **B.** Quantification of the location and format of GFP signals in the four core strains represented in panel A. The percentages of cells in panel A with APCs/ABCs (left), GFP particles in vacuoles (middle), and soluble GFP in vacuoles (right) were quantified for *vps21Δ*, *vps21Δvtc4Δ*, *vtc4Δ*, and *pep4Δ* cells starved in SD-N medium for different durations. Quantification was performed as done in Fig 2B. Over 200 cells were counted for each strain.

To further confirm the function of Vtc-protein depletion in promoting APC entry into vacuoles in *vps21Δ* cells, we studied Vtc4 depletion as an example. We intentionally grew *vps21Δ* cells to a low cell density (OD_600_ = 0.5) to accumulate more APs and inhibit autophagy on a large scale (Fig 1C and 1D). We also added nitrogen-starvation time points between 0 and 2 h and analyzed the effects by immunoblotting analysis. It is very clear that GFP-Atg8 entered vacuoles faster in *vps21Δvtc4Δ* cells than in *vps21Δ* cells under the same conditions. GFP-Atg8 dots started to appear in the vacuoles of *vps21Δvtc4Δ* cells even at 0 h of nitrogen starvation and were abundant in vacuoles at 1 h of nitrogen starvation. The *vps21Δvtc4Δ* cells appeared unable to accumulate GFP-Atg8 clusters outside vacuoles as occurred in *vps21Δ* cells. Instead, GFP-Atg8 particulates accumulated in vacuoles in *vps21Δvtc4Δ* cells, similar to *pep4Δ* cells. The *vtc4Δ* cells did not accumulate APCs but showed soluble GFP in vacuoles, similar to WT cells (Fig 8). Immunoblotting analysis showed that GFP-Atg8 degradation and Ape1 maturation in *vps21Δ* and *vps21Δvtc4Δ* cells were effectively inhibited when the cells had a low initial cell density before nitrogen starvation. A slight increase of GFP-Atg8 processing was repeatably observed in *vps21Δvtc4Δ* cells, but the degree of processing was not significantly different than that in *vps21Δ* cells (S3B and S3C Fig). These results indicate that most Vtc proteins negatively regulated the entry of unclosed APCs into vacuoles.

The fusion of closed mature APs with vacuoles requires the Rab GTPase Ypt7, the tether HOPS, and the SNARE protein Vam3 [31]. We confirmed that the absence of Ypt7 and the HOPS-specific subunits Vps39 and Vps41 generated similar autophagy defects [18]. To further test whether the entry of unclosed APs into vacuoles in *vps21Δ* cells also requires these proteins, we deleted *YPT7* or *VAM3* from WT and *vps21Δ* cells, after which we examined GFP-Atg8 localization and autophagy processing. *YPT7* deletion disrupted the vacuolar morphology and affected the GFP-Atg8 phenotype in *vps21Δ* cells. Since *ypt7Δ* and *vps21Δypt7Δ* cells had similar GFP-Atg8 phenotypes and vacuolar morphologies and because previous data showed that GFP-Atg8 in *ypt7Δ* cells did not enter fragmented vacuoles in *ypt7Δ* cells ([7] and S4A Fig), we speculated that GFP-Atg8 in *vps21Δypt7Δ* cells also did not enter the fragmented vacuoles. Our immunoblotting results showed that *YPT7* deletion completely blocked the partial-autophagy process (GFP-Atg8 degradation and prApe1 maturation) in *vps21Δ* cells, similar to the results in *ypt7Δ* cells (S4B and S4C Fig). These data suggest that the entry of unclosed APs into vacuoles required Ypt7 in *vps21Δ* cells. Depleting Vam3 from *vps21Δ* cells also resulted in decreased autophagy (S4A–S4C Fig), indicating that Vam3 was only partially required for unclosed APs to enter vacuoles but fully required for closed APs to enter vacuoles. Together, our data suggest that both Ypt7 and Vam3 were required for unclosed APs to enter vacuoles.

### The physiological relevance of the entry of unclosed APCs into vacuoles in *vps21Δ* cells during nitrogen starvation

Yeast autophagy is essential for starvation resistance and sporulation. The absence of core autophagy-machinery proteins, such as Atg1, resulted in decreased cell viability under nitrogen starvation in yeast haploid cells and infertility in diploid cells [14,15]. Starvation resistance and sporulation assays often take a few days of monitoring, whereas accumulated APCs in *vps21Δ* cells started to enter vacuoles between 2 and 8 h (even in *vps21Δpep4Δprb1Δ* cells) and most accumulated APCs ultimately entered vacuoles. It is impossible to compare transient physiological differences between *vps21Δ* cells containing APCs outside vacuoles after approximately 2 h of nitrogen starvation and those containing APCs inside vacuoles after approximately 8 h of nitrogen starvation using either of these assay methods.

However, if autophagy was completely blocked because the unclosed APCs could not enter vacuoles for degradation in *vps21Δ*/*vps21Δ* (diploid) cells, then the cells would not sporulate, just like *atg1Δ*/*atg1Δ* and *pep4Δ*/*pep4Δ* cells, in which autophagy was not initiated or fulfilled, respectively. Otherwise, if *vps21Δ*/*vps21Δ* cells were not infertile as *atg1Δ*/*atg1Δ* and *pep4Δ*/*pep4Δ* cells were under sporulation conditions, then it would be certain that autophagy in *vps21Δ*/*vps21Δ* cells was not completely blocked (i.e., the accumulated APCs in *vps21Δ*/*vps21Δ* cells entered vacuoles resulting in proliferation).

**Fig 9:**
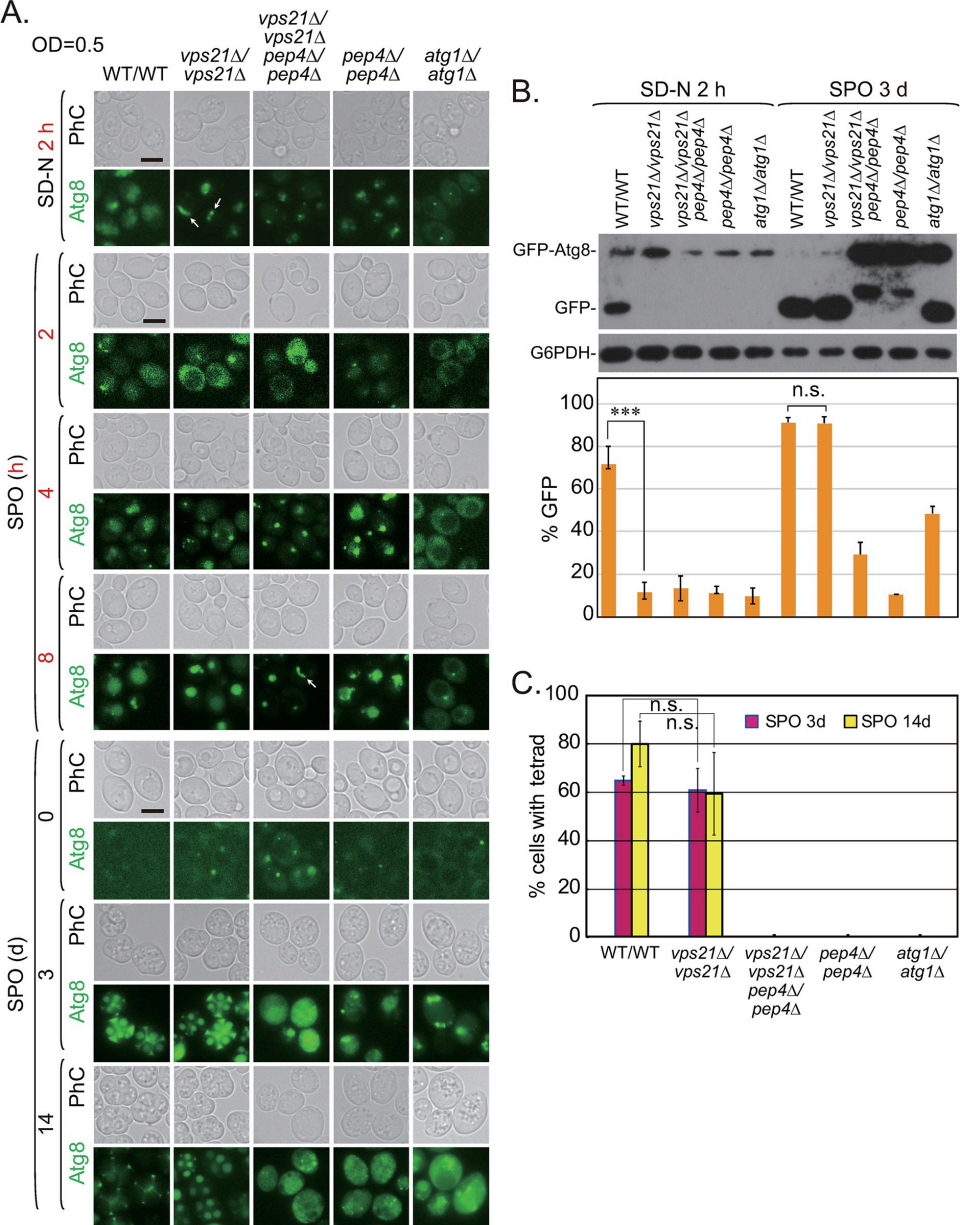
Diploid *vps21Δ*/*vps21Δ* cells could sporulate in sporulation medium (SPO). **A.** The *vps21Δ*/*vps21Δ* cells were capable of sporulation like WT/WT cells. The indicated diploid cells were grown in SD-N medium for 2 h (top), SPO for different numbers of h in the first day (middle), or SPO for days (bottom), after which PhC and GFP fluorescence images were obtained. GFP-Atg8-labeled clusters clearly accumulated in *vps21Δ*/*vps21Δ* cells in SD-N medium. Scale bars, 5 μm; arrows, APCs. **B.** Immunoblotting analysis of the autophagy process in the indicated diploid cells grown in SD-N medium for 2 h (left) or SPO for 3 days (right). The cells were grown as described in panel A and collected for immunoblotting analysis as described in Fig 1B. **C.** The percentages of cells with tetrad spores shown in panel A after sporulation proceeded for 3 and 14 days. n.s., not significant; *p < 0.05; ***p < 0.001. The results shown represent at least two independent experiments.

We examined the sporulation ability of *vps21Δ/vps21Δ* cells, with WT/WT, *pep4Δ*/*pep4Δ*, and *atg1Δ*/*atg1Δ* cells used as controls. In *vps21Δ/vps21Δ* cells, GFP-Atg8 also accumulated strongly in APCs after 2 h of nitrogen starvation (Fig 9A, top), and GFP-Atg8 degradation was completely blocked (Fig 9B). Autophagy was not efficiently induced after growth in sporulation medium (SPO) for 2 h with all tested cell lines. However, 4 or 8 h was sufficient to induce autophagy in all tested cells except for *atg1Δ*/*atg1Δ* cells. GFP-Atg8 entered vacuoles in WT/WT cells. Puncta but not APCs were observed in *vps21Δ/vps21Δ* and *vps21Δ/vps21Δ pep4Δ/pep4Δ* cells grown in SPO for 4 h. The puncta disappeared in *vps21Δ/vps21Δ* cells although puncta or APCs were observed in *vps21Δ/vps21Δ pep4Δ/pep4Δ* cells grown in SPO for 8 h (Fig 9A, middle). When the diploid cells were grown in SPO for 3 days, tetrads were observed in over 60% of WT/WT cells but not in *pep4Δ*/*pep4Δ* and *atg1Δ*/*atg1Δ* cells. Tetrads were easily observed in approximately 60% of *vps21Δ*/*vps21Δ* cells. GFP signals regularly distributed in the spores and ascal cytosol of tetrads. The percentage of WT/WT cells with tetrads increased to approximately 80% after growth in SPO for 14 days, whereas no significance was found regarding the percentage of *vps21Δ*/*vps21Δ* cells with tetrads (Fig 9A and 9C). After growth in SPO for 14 days, the GFP signals in tetrads decayed more in WT/WT cells than in *vps21Δ*/*vps21Δ* cells, whereas GFP-Atg8 degradation was almost complete in both WT/WT and *vps21Δ*/*vps21Δ* cells after growth in SPO for 3 days (Fig 9A and 9B). The sporulation ability was completely abolished in *vps21Δ*/*vps21Δ pep4Δ*/*pep4Δ* cells (Fig 9A and 9C). Although the combination of *pep4Δ*/*pep4Δ* should delay the entry of unclosed APCs into vacuoles in *vps21Δ*/*vps21Δ* cells, the unclosed APCs ultimately entered vacuoles and were not degraded due to the absence of the vacuolar hydrolase Pep4. GFP-Atg8 degradation in *vps21Δ*/*vps21Δ pep4Δ*/*pep4Δ* cells was similar to that in *pep4Δ*/*pep4Δ* cells (Fig 9B). The partial degradation of GFP-Atg8 in sporulation-defective strains (such as the *atg1Δ*/*atg1Δ* and *pep4Δ*/*pep4Δ* strains) grown in SPO for 3 days did not induce sporulation (Fig 9 and [32]). We propose that Atg1-independent proteolysis occurs that may be proceeded by vacuolar hydrolases when cells are grown in SPO. This possibility should be investigated in the future. Our results indicate that the entry of unclosed APCs into vacuoles in *vps21Δ*/*vps21Δ* cells and their complete degradation with vacuolar hydrolases led to sporulation; otherwise, they should have behaved like the infertile *atg1Δ*/*atg1Δ* and *pep4Δ*/*pep4Δ* cells.

### Depleting of vacuolar hydrolases exacerbates unclosed APC accumulation around vacuole membranes in ESCRT mutants, and APCs enter vacuoles after prolonged nitrogen starvation

Unclosed APCs accumulated around vacuole membranes in most cells of ESCRT- subunit mutants [10]. We wondered whether those APCs might enter vacuoles after prolonged nitrogen starvation. Thus, we observed GFP-Atg8 and FM4-64 fluorescence in representative ESCRT mutants (*snf7Δ* cells and *vps4Δ* cells) lacking two key vacuolar hydrolases (Pep4 and Prb1) after 2 h of nitrogen starvation. We confirmed that increased APC accumulation occurred in ESCRT mutants lacking both vacuolar hydrolases (S5A Fig and [10]). As expected, the partial GFP-Atg8 degradation and prApe1 maturation in ESCRT-mutant cells were impaired when *PEP4* and *PRB1* were further deleted (S5B Fig). We also examined GFP-Atg8 and FM4-64 localizations in *snf7Δ* and *snf7Δpep4Δprb1Δ* cells after prolonged nitrogen starvation. We found that the percentage of cells with GFP-Atg8 clusters in *snf7Δpep4Δprb1Δ* cells was significantly higher than that in *snf7Δ* cells between 2–6 h of nitrogen starvation. APCs in *snf7Δ* and *snf7Δpep4Δprb1Δ* cells gradually entered vacuoles between 2–6 h, and this phenomenon occurred faster in *snf7Δ* cells (S5C and S5D Fig). The GFP-Atg8-labeled APCs also gradually entered vacuoles in *vps4Δ* cells after prolonged nitrogen starvation. It is noteworthy that not all APCs entered vacuoles and became completely degraded, as ~40% of GFP-Atg8 and ~40% of prApe1 in *vps4Δ* cells were not processed even after 24 h of nitrogen starvation (S6 Fig), although more time might have been needed for the degradation to occur.

Ultrastructural analysis by TEM showed that AP-related membrane structures appeared inside vacuoles in ESCRT-mutant cells or cells further lacking Pep4 and Prb1 after 8 h of nitrogen starvation, where a significantly higher percentage of cells displayed APCs inside vacuoles than outside vacuoles (S7A and S7B Fig). Protease-protection assays showed that these AP-related membrane structures were unclosed in ESCRT-mutant cells or cells further lacking Pep4 and Prb1 after either 2 or 8 h of nitrogen starvation (S7C and S7D Fig). These results were similar to those in *vps21Δ* and *vps21Δpep4Δprb1Δ* cells under the same conditions (Figs 2, 3 and 6). Therefore, we propose that AP-related membrane structures inside vacuoles of ESCRT-mutant cells or cells further lacking Pep4 and Prb1 were unclosed single-membrane ABs converted from unclosed double-membrane APCs outside vacuoles.

### Overexpression of vacuolar hydrolases promotes the entry of unclosed APCs into vacuoles in ESCRT-mutant cells but does not lead to AP closure

Vacuolar hydrolases are required for unclosed APCs to enter vacuoles in Vps21 mutants. Here we investigated whether their overexpression could drive the entry of APCs into vacuoles in ESCRT mutant cells. Fluorescence microscopy data showed that Prb1 overexpression decreased the percentage of *snf7Δ* and *vps4Δ* cells displaying APCs while increasing vacuolar GFP signals (S8A and S8B Fig). The results of GFP-Atg8 degradation were consistent with the fluorescent microscopy observations, i.e., GFP-Atg8 degradation was enhanced in *snf7Δ* and *vps4Δ* cells when Prb1 was overexpressed (S8C and S8D Fig). Another hydrolase, Pep4, also suppressed the autophagic defects in ESCRT-mutant cells (S9A–S9D Fig).

ESCRT subunits were previously shown to seal the phagophores in the corresponding ESCRT mutants, both *in vivo* and *in vitro* [10]. Overexpressing vacuolar hydrolases (Prb1 and Pep4) partially suppressed APC accumulation in ESCRT-mutant cells and promoted autophagy, similar to the phenotypes observed after overexpressing ESCRT subunits in ESCRT-mutant cells. Most likely, overexpressing vacuolar hydrolases does not promote phagophore sealing in ESCRT-mutant cells. To clarify this issue, we performed protease-protection assays after overexpressing Snf7 and Prb1 in *snf7Δ* cells as an example. The “pellet” and “pellet + 1/3 supernatant” sample sets were prepared as described in the Materials and Methods section. The protease-protection assay results for the “pellet” set showed that Snf7 sealed phagophores in *snf7Δ* cells, but Prb1 did not, whereas the results for the “pellet + 1/3 supernatant” set showed that both Snf7 and Prb1 promoted autophagy in *snf7Δ* cells but only Snf7 sealed phagophores in *snf7Δ* cells (S8E and S8F Fig). Similarly, we found that both Snf7 and Pep4 promoted autophagy in *snf7Δ* cells but that only Snf7 sealed phagophores in *snf7Δ* cells (S9E and S9F Fig). These results indicate that the elevated level of autophagy in ESCRT-mutant cells after overexpressing vacuolar hydrolases was not due to an effect on phagophore sealing.

## Discussion

**Fig 10:**
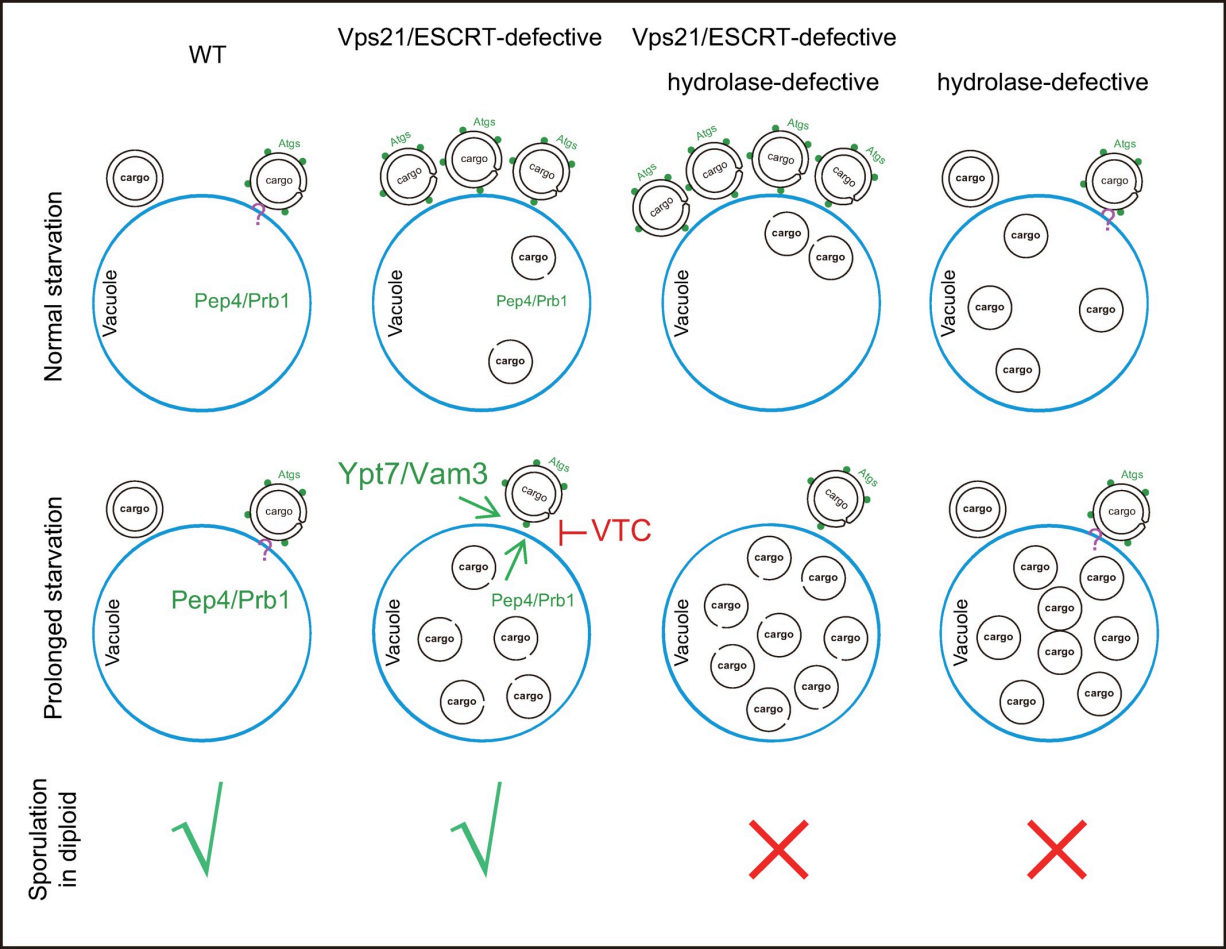
Diagram of the promotion of unclosed autophagosome entry into vacuoles in Vps21/Rab5- and ESCRT-mutant cells after prolonged nitrogen starvation. The entry of unclosed double-membrane autophagosomes into vacuoles after prolonged nitrogen starvation required Vam3 and Ypt7, was negatively promoted by VTC proteins, and was associated with the vacuolar hydrolases Pep4 and Prb1. However, it remains unclear how the unclosed double-membrane autophagosomes entered vacuoles to become unclosed single-membrane structures (autophagic bodies), which are sensitive to protease digestion, and are dissociated with Atg11. It also remains unknown whether unclosed autophagosomes in WT and hydrolase-defective mutants enter vacuoles. However, the entry of unclosed autophagosomes into vacuoles in diploid Vps21-mutant cells was clearly beneficial for sporulation.

Our previous findings showed that unclosed double-membrane APs (phagophores) accumulated outside vacuoles in Vps21- and ESCRT-mutant cells shortly after nitrogen starvation or rapamycin treatment [10,11]. Here we found that most APCs in Vps21- and ESCRT-mutant cells entered vacuoles and eventually became unclosed single-membrane ABs after prolonged nitrogen starvation. We also showed that the APCs in Vps21-mutant cells entered vacuoles after prolonged rapamycin treatment. The unclosed single-membrane ABs in vacuoles underwent partial degradation if vacuolar hydrolases were at suitable levels. Depleting vacuolar hydrolases slowed down the entry process for APs, whereas overexpressing vacuolar hydrolases potentially facilitated the entry process through more efficient degradation of APs inside the vacuoles; the depletion of most VTC proteins might facilitate the entry process through some other unknown mechanism(s). The entry of unclosed APs into vacuoles required Ypt7 and Vam3. The detailed mechanism underlying the entry of both closed and unclosed APs into vacuoles awaits further investigation. Importantly, the entry of unclosed APCs into vacuoles in diploid Vps21-mutant was beneficial for sporulation (Fig 10). Our results open avenues for further investigating the fate of unclosed APs that reside outside of vacuoles after prolonged autophagy induction.

### Unclosed double-membrane APs enter vacuoles to become single-membrane ABs

Double-membrane APs outside vacuoles and single-membrane ABs inside vacuoles were not clearly detected by conventional TEM in most yeast studies [6,7,25]. However, a hypothesis was proposed that the outer membrane of APs fused with the vacuole membrane to release APs only with an inner membrane and the contents as single-membrane ABs [6], and this proposal has been widely accepted by researchers in autophagy field. If this hypothesis is correct, then many questions remain unresolved. For example, the total surface area of ABs inside vacuoles in yeast hydrolase-defective mutants (such as *pep4Δprb1Δ* in Figs 3A and S7A) can easily exceed the vacuolar surface area, as ABs can fill an entire vacuole as autophagy proceeds. This means that the vacuole membrane would be occupied by the outer AP membrane quickly, unless the vacuole membrane was rapidly regenerated. In fact, the fusion of only the outer AP membrane (but not the inner AP membrane) with lysosome/vacuole membranes was not dynamically demonstrated in live cells either in yeast or mammalian cells.

Moreover, TEM and protease-protection assays have revealed that the APs in *ypt7Δ* cells are closed double-membrane structures in the cytoplasm before they can fuse with vacuoles [7]. Therefore, it is generally believed that only closed double-membrane APs can fuse with vacuoles for entry. TEM is a straightforward approach for observing phagophores (unclosed double-membrane APs) in mammalian and plant cells given that their APs are relatively large and unclosed APs have a crescent-shaped morphology [33,34]. In contrast, observing phagophores in yeasts and determining whether the outer membrane of phagophores fuse with vacuoles by TEM is challenging. The APs and phagophores of yeast cells are relatively small, and no method has been developed to morphologically distinguish between unclosed APs and closed APs if the unclosed hole is relatively small. Furthermore, autophagy is a continuous process with phagophores and closed APs co-existing in the same cell. When closed APs represent the main components of the pool of APs and phagophores, even the protease-protection assays cannot reveal whether phagophores are present in the pool because the results obtained with closed APs predominate over those obtained with phagophores. Therefore, it remains unknown whether phagophores (unclosed double-membrane APs) enter vacuoles like closed double-membrane APs do.

If an accumulation of unclosed double-membrane APs outside vacuoles could be created and conditions to induce the entry of these APs into vacuoles could be established, it would be possible to show the entry process. We overcame the current difficulties in studying yeast phagophore entry into vacuoles by utilizing three key methods in this study. First, we intentionally used Vps21- or ESCRT-mutants with depleted vacuolar hydrolases to (i) accumulate the maximum number of unclosed APCs outside vacuoles shortly after initiating nitrogen starvation and (ii) to block their degradation inside vacuoles for visualization purposes after extending the nitrogen-starvation time to induce the entry (Figs 1–3, S5 and S7). Thus, this method achieved two desired outcomes. Second, we applied time-lapse microscopy to provide **direct** evidence supporting the entry of unclosed double-membrane APs into vacuoles (Fig 5 and S7 Movie). Third, we clearly showed the single-membrane characteristic of APs after they entered vacuoles by Cryo-FIB and Cryo-ET analysis (Fig 4). Indeed, similar clear images showing the single-membrane characteristic of ABs and double-membrane characteristic of APs or phagophores (obtained using the same techniques) were reported in a bioRxiv reprint during the final preparation of this manuscript [35].

Additionally, the conventional and modified protease-protection assays and Atg11-dissociation assays illustrated that the APCs outside vacuoles and ABs inside vacuoles in these mutant cells were unclosed. These data strongly support the possibility that unclosed double-membrane APs outside vacuoles enter vacuoles to become unclosed single-membrane ABs. These observations in Vps21- and ESCRT-mutants do not represent a simple delay of AP closure or the general autophagy process, considering that overexpressing hydrolases in ESCRT-mutants promoted AP entry into vacuoles when they were still open (S8E, S8F, S9E and S9F Figs). Instead, our findings reflect a delay in entering into vacuoles for unclosed APs with double membranes, **not a delay in closing** unclosed APs before or after they enter vacuoles. To our knowledge, this is the first report to show the entry process of phagophores into vacuoles and to show unclosed single-membrane ABs inside vacuoles under prolonged nitrogen starvation.

Autophagy is a highly conserved cellular-degradation and recycling system that serves essential cell-survival roles under adverse conditions and directs many other functions [36]. The fusion of closed APs with vacuoles should be favorable in WT yeast cells when autophagy is induced. However, it remains unknown whether phagophores enter vacuoles during a short period of nitrogen starvation. Even WT cells might undergo increased autophagic flux under extreme conditions. It is also unknown whether unclosed APs fuse with vacuoles for recycling under extreme conditions. Importantly, unfavorable conditions might occur often in nature, and the yeast cells might experience much longer nitrogen-starvation periods in nature than the usual 2–4 h of nitrogen starvation in a laboratory setting.

Our current finding that unclosed APCs entered into vacuoles suggests an alternative means whereby cellular contents can be recycled. This is especially important for mutants that accumulate abnormal levels of unclosed APs; otherwise, these cells might behave like the core autophagy-machinery mutants, which showed poor survival and proliferation under unfavorable conditions (Fig 9). Enhancing autophagy by (i) increasing the cell density before nitrogen starvation or (ii) overexpressing vacuolar hydrolases did not clearly increase the resistance to starvation by the Vps21- and ESCRT-mutant cells, most likely because using a long nitrogen starvation time (1 day to a few days) before examining the cell viabilities reduced their differences in autophagy. However, our findings with *vps21Δ*/*vps21Δ* cells suggest that the entry of unclosed APCs into vacuoles was beneficial for sporulation; otherwise, the cells would have been infertile like the *atg1Δ*/*atg1Δ* and *pep4Δ*/*pep4Δ* cells (Fig 9). The additional physiological relevance of the entry of unclosed APCs into vacuoles should be investigated further in the future.

### Regulation of the entry of unclosed APCs into vacuoles

The fusion of the outer membrane of closed APs with vacuole membrane was not observed dynamically in live cells, and the related mechanism remains unclear. However, it is known that the fusion of closed APs with vacuoles requires Ypt7 and Vam3 [31]. An important question is how the entry of unclosed double-membrane APs into vacuoles is regulated.

This study was motivated by our observations that the growth-stage before nitrogen starvation and the availability of vacuolar hydrolases greatly affected the percentage of Vps21- and ESCRT-mutant cells displaying accumulated APCs after 2 h of nitrogen starvation (Fig 1 and S5A Fig). The important roles of vacuolar hydrolases in affecting the entry of unclosed APCs into vacuoles were supported by depleting or overexpressing Pep4 and Prb1 (Figs 1E–1F, 2, 3, 7, S5, S7A, S7B, S8A–S8D and S9A–S9D). Depleting vacuolar hydrolases in Vps21- and ESCRT- mutant cells slowed down the entry of unclosed APCs into vacuoles. Overexpressing vacuolar hydrolases in these mutant cells might facilitate the entry process through more efficient APC degradation inside vacuoles. This is because the unclosed APCs that accumulated outside vacuoles in these mutant cells need to enter vacuoles before they are degraded by vacuolar hydrolases, which rely on the acidic environment inside vacuoles for activity. If the APCs did not enter vacuoles, then more efficient degradation of other cargo molecules/substrates (but not APCs) would result from overexpressing a protease, and the percentage of cells displaying APCs would not decrease. Furthermore, the growth-stage dependent increase of vacuolar hydrolases [12,13,37–39] helps explain the decreased APC accumulation in cells starting from a high cell density before nitrogen starvation. These findings mean that feedback might occur from the degradation in vacuoles and direct the entry of accumulated APCs into vacuoles. Enhanced APC degradation inside vacuoles enables more APCs to enter vacuoles. We do not have any evidence indicating that the lumenal proteases (Pep4 and Prb1) play a direct role in the fusion of unclosed autophagosomes with vacuoles. The observed phenotypes might have been due to indirect effects. The hypothesized feedback-regulation mechanism and the real roles of proteases in the entry of APCs into vacuoles need to be investigated in the future.

We found that individually depleting VTC proteins (Vtc1, 2, 4, or 5) promoted the entry of unclosed APCs into vacuoles in *vps21Δ* cells, which partially agrees with the functions of the Vtc1-4 proteins in regulating the entry of misfolded glycosylphosphatidylinositol-anchored proteins stacked on vacuole membranes in *pep4Δ* cells into vacuoles [30]. The underlying mechanisms for both processes require further investigation. In terms of positive regulators, we found that the entry of unclosed APs into vacuoles also required Ypt7 and Vam3 (S4 Fig), suggesting that the steps mediating the entry of both unclosed and closed APs into vacuoles involve the same or similar machinery. When abnormal APs were available to enter vacuoles in the mutant cells, the machinery might not be able to distinguish whether the APs were closed or unclosed, or the machinery might identify the difference between closed or unclosed APs but drive the entry of available unclosed APs into vacuoles less efficiently.

Taken together, these findings suggest that APC entry into vacuoles depends on the enhanced degradation of autophagic contents in vacuoles, which is affected by levels of vacuolar hydrolases. In addition, most VTC proteins delayed the entry of unclosed APCs into vacuoles, and Ypt7 and Vam3 promoted such entry, although the existence of more unknown negative and positive regulators cannot be excluded. The detailed regulatory mechanism remains to be investigated in the future.

### Limitations of this study and future directions

Although the entry of unclosed double-membrane APs into vacuoles after prolonged nitrogen starvation and the subsequent formation of unclosed single-membrane ABs inside vacuoles were demonstrated in this study, it remains unclear how double-membrane APs were converted to single-membrane ABs regardless of whether the APs were closed or unclosed. Fusion of the outer membrane of closed APs with the membranes of vacuoles/lysosomes to release APs only with an inner membrane and contents as single-membrane ABs was proposed [6], but this dynamic process has not been proven. This issue was still not resolved in this study for both closed and unclosed APs and will remain unclear until suitable techniques are developed in the future. Furthermore, the unclosed double-membrane APs in Vps21- and ESCRT-mutant cells must exist an unobserved open pore facing the cytosol. Similarly, unclosed single-membrane ABs in vacuoles may contain such an unobserved open pore facing the vacuolar lumen. However, it may be challenging to maintain an open pore in an unclosed single membrane because such pores are thought to be unstable. It is unknown whether caps exist at the edges of pores in unclosed single-membrane ABs. Another concern regarding the formation of unclosed single-membrane ABs is that it is unclear how the inner membrane was cut off from the outer membrane of an unclosed AP and still maintained a pore. If the size of the pore of the unclosed single-membrane AB is sufficiently small, the morphology of the unclosed single-membrane AB might be stable for a long time. However, it remains unknown how such a cut-off might be realized and regulated. In addition, no evidence exists showing that the fusion of unclosed APs with vacuoles is an important process in WT cells. We propose that APs preferentially fuse with vacuoles through the conventional mechanism in WT cells, i.e., phagophores close to become mature APs, which then fuse with vacuoles, although the possibility that unclosed APs fuse with vacuoles cannot be excluded. No good way has been developed to distinguish between the unclosed and closed APs in WT cells or to track their fates accurately to date. Furthermore, it is unclear whether the entry of unclosed APs into lysosomes also occurred in mammalian cells as phagophores accumulated in mammalian ESCRT-mutant cells [33].

In summary, herein we report a novel autophagic process where unclosed double-membrane APs enter vacuoles under prolonged autophagy induction in budding yeast, which is important for sporulation. This process might involve the same molecular machinery that enables closed mature APs to enter vacuoles. However, the underlying mechanism and additional physiological relevance of this process, whether this process occurs in normal cells, and the conservation of this process in plant and mammalian cells need to be explored in the future.

## Materials and methods

### Strains, plasmids, and reagents

The yeast strains and plasmids used in this study are indicated in S1 Table. Deletion strains in the haploid state were obtained by replacing the complete open-reading frame of the targeted genes with amino acid or drug cassette encoding genes using PCR-based homologous recombination with strains developed in previous studies [10,11,18]. A Vph1 with a GFP tagged at the C-terminal end was constructed by PCR-based homologous recombination as described [11]. *PRB1* or *PEP4* was cloned into the pRS415 vector and expressed under the *ADH1* promoter to obtain pRS415-*ADH1p-PEP4* or pRS415-*ADH1p-PRB1*.

All yeast and *Escherichia coli* transformations were performed as described [40]. The antibodies and reagents used in this study were described previously [11].

### Yeast culture conditions and autophagy induction

Cells cultured overnight in YPD or selective dropout medium (SD) were inoculated at an OD_600_ of ~0.05 and grown for ~8 h to mid-log phase at 26°C in YPD or selective medium, re-inoculated at an OD_600_ of ~0.05, and grown to an OD_600_ of ~0.5, as described [11]. Then, the cells were treated with 10 nM rapamycin or shifted from YPD medium to SD-N medium (without amino acids and ammonium sulfate) for starvation at 26°C for the indicated number of h and subjected to different assays, as described below. To determine the effect of cell density of the culture before nitrogen starvation on autophagy induction, the cells were grown to different OD_600_ values (0.5–4) in YPD medium and readjusted to a similar OD_600_ value of ~0.8–1 in SD-N medium for nitrogen starvation. For vacuole staining, 1.6 μM of FM4-64 was added to the medium 1 h before the cells were harvested as described [11].

### Live-cell microscopy and time-lapse imaging

Cells expressing fluorescent proteins or stained by FM4-64 were examined using a Nikon Eclipse Ti inverted research microscope, a Leica LAS X confocal microscope, or an UltraVIEW spinning-disk confocal scanner unit (PerkinElmer, Waltham, MA) as described [11]. More than three fields were visualized for each sample. The data were quantified as indicated in the figure legends. For time-lapse imaging, cells growing in mid-log phase were shifted to SD-N medium to induce autophagy for 1 h and loaded onto a solid SD-N-medium pad on a glass slide for observation. Images were captured at a fixed rate of 1 min per timepoint with two channels (488 nm for GFP and 561 nm for mCherry) and three Z-stacks (step size of 0.5 μm) using an UltraVIEW spinning-disk confocal scanner unit for about 5 h. The GFP and mCherry frames at 10-minute intervals of the movie were shown as still images in Fig 5C.

### Autophagy assay

Autophagic cargo processing was studied via immunoblotting analysis using anti-GFP and anti-Ape1 antibodies to detect GFP-Atg8 and prApe1 processing, respectively, as described [11].

### TEM analysis

Cells were grown, processed, and quantified for TEM analysis as described [11].

### Cryo-FIB and Cryo-ET analyses

Cells were grown to an OD_600_ of ~0.5 as described above, starved in SD-N medium for 2 or 8 h, and applied to fresh glow-discharged molybdenum EM finder grids (with lacey carbon), blotted on a single side (opposite to the side the cells were on) using an EM GP2 plunge freezer (Leica), and plunged into ethane that was precooled with liquid nitrogen. The grids with vitrified yeasts were clipped and loaded into a Helios NanoLab 600i DualBeam FIB/SEM instrument. The samples were coated with platinum for better conductivity. Currents of 0.43 nA and 0.23 nA were used for rough milling, and a lower current of 80 pA was used for the polishing step to obtain the final lamellas (approximately 150 nm thick) [41]. The lamellas were imaged using a 300 kV Titan Krios-electron microscope (Thermo Fisher Scientific) equipped with a field mission gun, a direct electron detector (Gatan, K2 camera), and an energy filter (Gatan). Tilt-series were collected from -42° to 60° at 3° increments using SerialEM software [42] in counting mode, with a total accumulated dose of 100 e−/Å^2^, at a defocus of -8 μm, an energy filter setting of 20 eV, and a final pixel size of 5.424 Å. Dose-fractioned images were aligned using MotionCor2 [43]. The corrected tilt-series were aligned using IMOD software [44] and reconstructed using the simultaneous iterative reconstruction technique implemented in the IMOD software package. Tomograms were 4× binned and used for visualization.

### Protease-protection assays

The conventional protease-protection assay was performed as described [11]. The modified microscopy-based protease-protection assay was performed as described [10]. The percentages of particles with GFP-Atg8 dots were quantified for all treatments of each strain. Then the absolute percentage of particles with GFP-Atg8 dots in samples without protease and detergent for each strain was set as the relative percentage of 100% to calculate the relative percentages of different treatments for the same strain. Either the APs in the cytosol or ABs inside vacuoles were isolated as particles for protease-protection analysis [10]–not as whole intact vacuoles (inferred by the western blot analysis of Prc1) together with ABs inside the vacuoles [45]. To test whether the overexpression of vacuolar hydrolase could seal the phagophores isolated from ESCRT-mutant cells, we prepared two parallel sets of cells. One set of cells was used to collect the pellet (AP-related membranes) as described [10,11]. The other set of cells was handled similarly except that 1/3 of supernatant obtained after the spheroplasting and spinning steps was pooled together with pellet (the “pellet + 1/3 supernatant” set).

### Sporulation assay

SEY6210 and SEY6210.1 strains with opposite mating types [46] were applied for sporulation assays. Target genes were deleted from SEY6210 or SEY6210.1 strains expressing GFP-Atg8 from their chromosomes. The GFP-Atg8-positive SEY6210 and SEY6210.1 strains were mated to obtain WT/WT. Correspondingly, the same deletion mutants in the GFP-Atg8-positive SEY6210 and SEY6210.1 strains were mated to produce diploid mutants. The diploid cells were grown to log phase in rich medium and subjected to growth in SD-N medium or SPO as described [47] for different durations before fluorescence microscopy observations were made.

### Statistical analyses

Analysis of variance using SPSS Statistics (IBM) or TTEST analysis using Excel (Microsoft) was applied to determine statistical significances as described previously [11]. The significance of p value is represented as follows: n.s., not significant; *p < 0.05; **p < 0.01; ***p < 0.001. Numerical data that underlies graphs is provided in spreadsheet form as supporting information in S1 Data.

## Supporting information

S1 Fig The accumulated APCs in *vps21Δ* cells entered vacuoles after prolonged rapamycin treatment and the AP-related membrane structures in *vps21Δ* cells grown in SD-N for 2 h are sensitive to PK digestion. **A.** The accumulated GFP-Atg8-labeled APCs in *vps21Δ* cells gradually entered vacuoles after prolonged rapamycin treatment. The indicated GFP-Atg8-labeled *vps21Δ* cells were grown in YPD medium as described in Fig 1A to an OD_600_ of 0.5, after which they were treated with 0 or 10 nM rapamycin or starved in SD-N for the indicated durations for fluorescence observations. The SD-N treatment and 0 nM rapamycin treatment served as controls. FM4-64 was omitted in this experiment because it delayed APC induction by rapamycin and APC entry into vacuoles. Scale bar, 5 μm; arrows, APCs. **B.** Quantification of *vps21Δ* cells containing APCs represented in panel A. The percentage of APC-positive *vps21Δ* cells peaked after ~2 h of nitrogen starvation and declined after that under prolonged nitrogen starvation (left). The percentage of APC-positive *vps21Δ* cells peaked at ~6 h and declined after that under 10 nM of rapamycin treatment (right). The quantitative data are presented as the mean +/- STD. Over 270 cells were counted for each treatment. **C.** Autophagy processing in *vps21Δ* cells after rapamycin treatment increased with the treatment time. Cells were grown as described in panel A. GFP-Atg8 and prApe1 processing were determined for cell lysates as described in Fig 1B. G6PDH was detected as a loading control. GFP-Atg8 processing to GFP and prApe1 processing to mApe1 were quantified and are presented below the G6PDH blot. The quantitative data are presented as the mean +/- STD. n.s., not significant. **D.** The modified microscopy-based PK-protection assay showed that GFP-Atg8 in AP-related membrane structures isolated from *vps21Δ* and *vps21Δpep4Δprb1Δ* cells grown in SD-N for 2 h were accessible to PK. The experiments were conducted as described in Fig 6C except that the cells were treated in SD-N for 2 h and one set of representative data from two repeats was presented. Scale bars, 2 μm; arrows point to GFP-Atg8-positive particles. GFP-Atg8 could not be observed in particles from *vps21Δ* and *vps21Δpep4Δprb1Δ* cells grown in SD-N for 2 h when PK is added to membranes without detergent. The right column indicates the total GFP-Atg8-positive dots in green numbers and particles in grey numbers from two independent experiments used for quantification. The results shown represent two independent experiments.
(TIF)

S2 Fig Atg11 was released from unclosed APCs in *vps21Δ* cells when they were inside vacuoles after prolonged nitrogen starvation. Strains expressing Atg11-GFP and mCherry-Atg8 from their chromosomes were grown and starved as reported [11] but with an additional nitrogen-starvation period of 8 h. Atg11-GFP colocalization with mCherry-Atg8 was monitored using live-cell fluorescence microscopy. The insets are from the frames for the merged pictures. The arrows indicate Atg11-Atg8 colocalization, and the arrowheads indicate cases where Atg8 did not colocalize with Atg11. Scale bars, 5 μm. Atg11 was removed from most Atg8-positive APs that accumulated in *ypt7Δ* cells before fusion or from most ABs that accumulated in *pep4Δ* cells after fusion, although Atg11 remained on most APCs on vacuole membranes that accumulated in *vps21Δ* cells after 2 h of nitrogen starvation (top and [10,11]). As APCs entered vacuoles in *vps21Δ* and *vps21Δpep4Δ* cells after 8 h of nitrogen starvation to become ABCs, most Atg11 detached from the ABCs. The results shown represent two independent experiments.
(TIF)

S3 Fig Most vacuolar transport chaperon (VTC) proteins inhibited APC entry into vacuoles in *vps21Δ* cells under nitrogen starvation. **A.** *VTC1-5* were individually deleted from GFP-Atg8-labeled WT and *vps21Δ* cells to generate the indicated strains. The strains were grown and starved in SD-N medium as described in Fig 2A for microscopic observations. Except for Vtc3, the depletion of all other Vtc proteins promoted the entry of accumulated APCs into vacuoles in *vps21Δ* cells under nitrogen starvation. Scale bars at 5 μm; arrows, APCs. **B.** Immunoblotting assays showing that partial autophagy processing in *vps21Δ* cells after nitrogen starvation increased slightly with the depletion of Vtc4. Cells were grown as described in Fig 8A, and autophagy processing was determined as described in Fig 1D. **C.** The autophagy process (% GFP and % mApe1) was quantified after 8 h of SD-N treatment for the samples represented in panel B. The quantitative data are presented as the mean +/- STD. n.s., not significant. The results shown represent two independent experiments.
(TIF)

S4 Fig Ypt7 and Vam3 were involved in the entry of unclosed APCs into vacuoles after prolonged nitrogen starvation. **A.** The entry of GFP-Atg8-labeled APCs into vacuoles was impaired when Ypt7 or Vam3 was depleted. *YPT7* or *VAM3* was deleted from *vps21Δ* cells to obtain *vps21Δypt7Δ* or *vps21Δvam3Δ* cells, respectively. The cells were grown, starved, and examined as described in Fig 2A. Arrows, APCs; scale bar, 5 μm. **B.** Autophagy processing was completely blocked in *vps21Δypt7Δ* cells and partially blocked in *vps21Δvam3Δ* cells after prolonged nitrogen starvation. The cells were grown as described in panel A. GFP-Atg8 and prApe1 processing were determined for cell lysates by performing immunoblotting assays as described in Fig 1B. **C.** Quantification of the blots presented in panel B. Quantification was performed as described in Fig 1D. The results shown represent at least two independent experiments.
(TIF)

S5 Fig The accumulated APCs in ESCRT-mutant cells increased in the absence of hydrolases, and APCs entered vacuoles after prolonged nitrogen starvation. **A.** The accumulation of GFP-Atg8-labeled APCs in ESCRT-mutant (*snf7Δ*, *vps4Δ*) cells after nitrogen starvation increased in the absence of the vacuolar hydrolases Pep4 and Prb1. The cells were grown and starved as described in Fig 1E. The percentages of cells containing APCs were quantified and are presented below the merged pictures. **B.** Partial autophagy processing in ESCRT-mutant cells after nitrogen starvation was completely blocked in the absence of vacuolar hydrolases. The cells were grown as described in panel A and autophagy processing was determined and presented as described in Fig 1D. **C.** The accumulated APCs in *snf7Δ* and *snf7Δpep4Δprb1Δ* cells entered vacuoles after prolonged nitrogen starvation. The indicated cells expressing GFP-Atg8 (as shown in panel A) were grown and starved as described in Fig 2A for fluorescence observations. In panels A and C: arrows, APCs; scale bars, 5 μm. **D.** Quantification of the cells containing APCs and GFP signals in vacuoles shown in panel C. The percentage of cells containing APCs peaked after 4 h of nitrogen starvation and declined after that in *snf7Δ* and *snf7Δpep4Δprb1Δ* cells (top), whereas the percentage of cells containing GFP in vacuoles gradually increased in *snf7Δ* and *snf7Δpep4Δprb1Δ* cells (bottom) under prolonged nitrogen starvation. The quantitative data are presented as the mean +/- STD. *p < 0.05; **p < 0.01; n.s., not significant. The results shown represent at least two independent experiments.
(TIF)

S6 Fig Autophagy was defective in *vps4Δ* cells although prolonged nitrogen starvation partially promoted autophagy. **A.** The accumulated GFP-Atg8-labeled APCs in *vps4Δ* cells gradually entered vacuoles after prolonged nitrogen starvation. The indicated cells that expressed GFP-Atg8 were grown and starved as described in Fig 2A for fluorescence observations. FM4-64 staining was performed to label the vacuole membranes for 1 h before the cells were collected for fluorescence microscopy. Arrows, APCs; scale bar, 5 μm. **B.** Autophagy processing in *vps4Δ* cells increased after prolonged nitrogen starvation. The cells were grown as described in panel A and subjected to immunoblotting assays as described in Fig 1B. G6PDH was detected as a loading control. **C.** Quantification of the *vps4Δ* cells shown in panel A that contained APCs and GFP signals in their vacuoles. The percentage of *vps4Δ* cells containing GFP in their vacuoles gradually increased, whereas the percentage of cells containing APCs peaked at 2h and subsequently declined after prolonged nitrogen starvation. **D.** The autophagy process was still defective in *vps4Δ* cells although prolonged nitrogen starvation partially promoted autophagy. The bands in panel B were quantified and presented as done in Fig 1F. The quantitative data are presented as the mean +/- STD. **p < 0.01. The results shown represent two independent experiments.
(TIF)

S7 Fig The APCs that accumulated in ESCRT-mutant cells after 8 h of nitrogen starvation were unclosed membrane structures. **A.** The accumulated APCs in ESCRT-mutant cells entered vacuoles after 8 h of nitrogen starvation. Cells were grown and treated as described in S5A Fig, but after 8 h of nitrogen starvation, they were subjected to ultrastructural analysis as described in Fig 3A. Vac, vacuole; Nuc, nucleus; E, class E compartment; red asterisks, APs; yellow asterisks, ABs; black asterisks, closed ABs. **B.** Quantification of the cells shown in panel A based on the categories of APCs defined in Fig 3B. n.s., not significant; *p < 0.05; **p < 0.01; ***p < 0.001. Over 300 slices were counted for each strain. **C-D.** Protease-protection assays showed that the AP-related membrane structures that entered the vacuoles of ESCRT-mutant cells (with or without hydrolases after prolonged nitrogen starvation) were unclosed membrane structures. GFP-Atg8 (C) and prApe1 (D) on AP-related membrane structures isolated from ESCRT-mutant cells after 8 h of nitrogen starvation were sensitive to protease K digestion. The cells were grown as described in S5C Fig and analyzed as described in Fig 6. The *pep4Δprb1Δ* cells served as a control for closed ABs. ***p < 0.001. The results shown represent at least two independent experiments.
(TIF)

S8 Fig Prb1 overexpression decreased APCs in ESCRT-mutant cells after nitrogen starvation. **A.** The accumulation of GFP-Atg8-labeled APCs in ESCRT- mutant (*snf7Δ*, *vps4Δ*) cells decreased with Prb1 overexpression. The indicated cells were transformed with a Prb1-expression plasmid or the empty vector (pRS415, ∅), grown, and examined as described in Fig 7A. Scale bar, 5 μm; arrows, APCs. **B.** Quantifications of cells containing soluble GFP in vacuoles. The percentage of cells containing GFP in vacuoles (shown in panel A) was quantified as the mean +/- STD. Over 600 cells were counted for each strain. **C.** GFP-Atg8 degradation in ESCRT- mutant cells increased with Prb1 overexpression. The cells were grown as described in panel A and examined for GFP-Atg8 degradation as described in Fig 1. G6PDH was detected as a loading control. **D.** Quantification of GFP-Atg8 degradation, based on the immunoblot shown in panel C. The quantification was performed as described in Fig 1D, and the data are presented as the mean +/- STD. **E.** The facilitation of autophagy by Prb1 in *snf7Δ* cells was not due to phagophore closure, as Snf7 did in *snf7Δ* cells. Cells transformed with a Prb1-expression plasmid, an Snf7-expression plasmid, or the empty vector (pRS415, ∅) were grown as described in panel A, except that they were starved for 3 h. The “pellet” and “pellet + 1/3 supernatant” sample sets were prepared from the indicated cells as described in the Materials and methods section and subjected to protease-protection assay and immunoblotting assays, exactly as described in Fig 6A and 6B. Either Prb1 or Snf7 in *snf7Δ* cells facilitated GFP-Atg8 degradation and prApe1 maturation (bottom), but only Snf7 (not Prb1) facilitated phagophore closure in *snf7Δ* cells. The red arrows point to these key results. **F.** The blots shown in panel E were quantified as described in Fig 6A and 6B. P values in panels B, D, and F: n.s., not significant; *p < 0.05; **p < 0.01; ***p < 0.001. The results shown represent at least two independent experiments.
(TIF)

S9 Fig Pep4 overexpression decreased APCs in Vps21- and ESCRT-mutant cells after nitrogen starvation. **A.** The accumulation of GFP-Atg8-labeled APCs in mutant cells (*vps21Δ*, *snf7Δ*, and *vps4Δ*) slightly decreased with Pep4 overexpression. The indicated cells were transformed with a Pep4-expression plasmid or the empty vector (pRS415, ∅), grown, and examined as described in Fig 7A. Scale bar, 5 μm; arrows, APCs. **B.** GFP-Atg8 degradation increased in Vps21- and ESCRT-mutant cells with Pep4 overexpression. The cells represented in panel A were examined for GFP-Atg8 degradation as described in Fig 1. **C.** Quantification of the cells shown in panel A that contained soluble GFP in their vacuoles, presented as the mean +/- STD. Over 250 cells were counted for each strain. **D.** Quantification of GFP-Atg8 degradation shown in the immunoblots in panel B. GFP-Atg8 degradation shown in panel B was quantified as described in Fig 1D, and the results are presented as the mean +/- STD. **E.** The facilitation of autophagy by Pep4 in *snf7Δ* cells was not due to phagophore closure, as Snf7 did in *snf7Δ* cells. Cells transformed with an Snf7-expression plasmid, a Pep4-expression plasmid, or the empty vector (pRS415, ∅) were grown as described in panel A, except that they were starved for 3 h. The experiments were conducted as described in S8E Fig. Either Pep4 or Snf7 in *snf7Δ* cells facilitated GFP-Atg8 degradation and prApe1 maturation (bottom), but only Snf7 (not Pep4) promoted phagophore closure in *snf7Δ* cells. The red arrows point to these key results. **F.** Quantification of the immunoblot results shown in panel E was performed as described in S8F Fig. P values in panels C, D, and F: n.s., not significant; *p < 0.05; **p < 0.01; ***p < 0.001. The results shown represent at least two independent experiments.
(TIF)

S1 Table Yeast strains and plasmids used in this study. **A.** Strains. **B.** Plasmids.
(PDF)

S1 Movie A tomogram for *vps21Δ* cells starved in SD-N for 2 h. This tomogram is relevant to Fig 4B.
(AVI)

S2 Movie A tomogram for *vps21Δpep4Δprb1Δ* cells starved in SD-N for 2 h. This tomogram is relevant to Fig 4B.
(AVI)

S3 Movie A tomogram for *pep4Δprb1Δ* cells starved in SD-N for 2 h. This tomogram is relevant to Fig 4B.
(AVI)

S4 Movie A tomogram for *vps21Δ* cells starved in SD-N for 8 h. This tomogram is relevant to Fig 4B.
(AVI)

S5 Movie A tomogram for *vps21Δpep4Δprb1Δ* cells starved in SD-N for 8 h. This tomogram is relevant to Fig 4B.
(AVI)

S6 Movie A tomogram for *pep4Δprb1Δ* cells starved in SD-N for 8 h. This tomogram is relevant to Fig 4B.
(AVI)

S7 Movie Time-lapse images for the entry of mCherry-Atg8-labeled APs into Vph1-GFP-labeled vacuoles in *vps21Δ* cells after prolonged nitrogen starvation. The *vps21Δ* cells were grown to mid-log phase and incubated in SD-N medium for 1 h before they were loaded onto an SD-N-medium pad on a glass slide. Images were captured at a fixed rate of 1 min per timepoint with two channels (488 nm for GFP and 561 nm for mCherry) and three Z-stacks (step size of 0.5 μm) using an UltraVIEW spinning-disk confocal scanner unit for 5.5 h. The time-lapse sequence (10 fps) is shown. Note the entry of mCherry-Atg8-labeled APs into Vph1-GFP-labeled vacuoles. The GFP and mCherry frames at 10 min intervals of the movie are shown as still images in Fig 5C.
(AVI)

S1 Data All of the quantification data and analysis of significance differences in Figs 1–9 and S1–S9 Figs are presented in the dataset (Quantification data.zip). (ZIP)
