# Supplementary figures and images for: The entry of unclosed autophagosomes into vacuoles and its physiological relevance
Source: PLoS Genet. 2022 Oct 13;18(10):e1010431. doi: 10.1371/journal.pgen.1010431 (PMC9562215; doi:10.1371/journal.pgen.1010431)

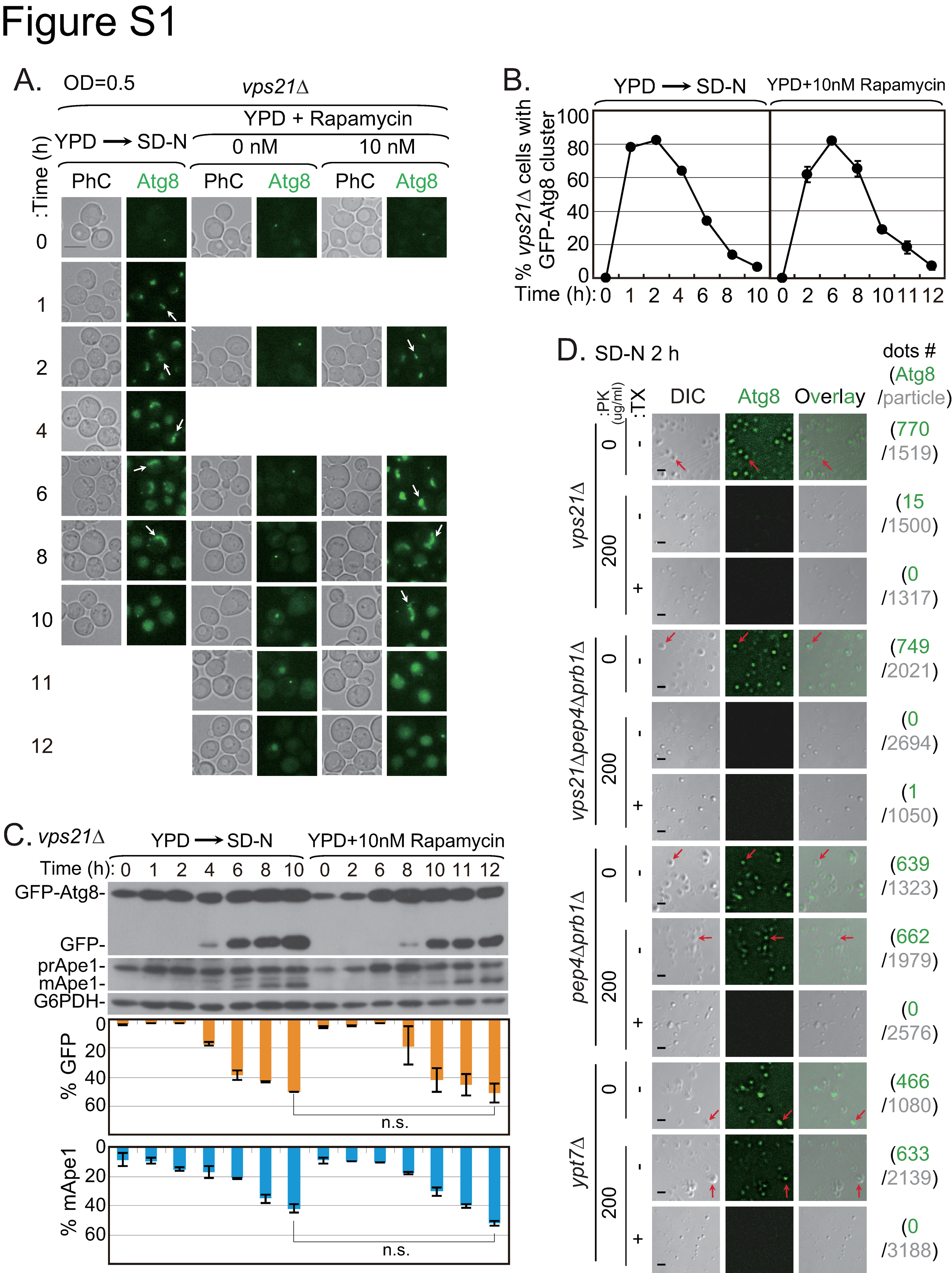

Supplement: S1 Fig — A. The accumulated GFP-Atg8-labeled APCs in vps21Δ cells gradually entered vacuoles after prolonged rapamycin treatment. The indicated GFP-Atg8-labeled vps21Δ cells were grown in YPD medium as described in Fig 1A to an OD600 of 0.5, after which they were treated with 0 or 10 nM rapamycin or starved in SD-N for the indicated durations for fluorescence observations. The SD-N treatment and 0 nM rapamycin treatment served as controls. FM4-64 was omitted in this experiment because it delayed APC induction by rapamycin and APC entry into vacuoles. Scale bar, 5 μm; arrows, APCs. B. Quantification of vps21Δ cells containing APCs represented in panel A. The percentage of APC-positive vps21Δ cells peaked after ~2 h of nitrogen starvation and declined after that under prolonged nitrogen starvation (left). The percentage of APC-positive vps21Δ cells peaked at ~6 h and declined after that under 10 nM of rapamycin treatment (right). The quantitative data are presented as the mean +/- STD. Over 270 cells were counted for each treatment. C. Autophagy processing in vps21Δ cells after rapamycin treatment increased with the treatment time. Cells were grown as described in panel A. GFP-Atg8 and prApe1 processing were determined for cell lysates as described in Fig 1B. G6PDH was detected as a loading control. GFP-Atg8 processing to GFP and prApe1 processing to mApe1 were quantified and are presented below the G6PDH blot. The quantitative data are presented as the mean +/- STD. n.s., not significant. D. The modified microscopy-based PK-protection assay showed that GFP-Atg8 in AP-related membrane structures isolated from vps21Δ and vps21Δpep4Δprb1Δ cells grown in SD-N for 2 h were accessible to PK. The experiments were conducted as described in Fig 6C except that the cells were treated in SD-N for 2 h and one set of representative data from two repeats was presented. Scale bars, 2 μm; arrows point to GFP-Atg8-positive particles. GFP-Atg8 could not be observed in particles from vps21Δ and [file pgen.1010431.s001.tif]

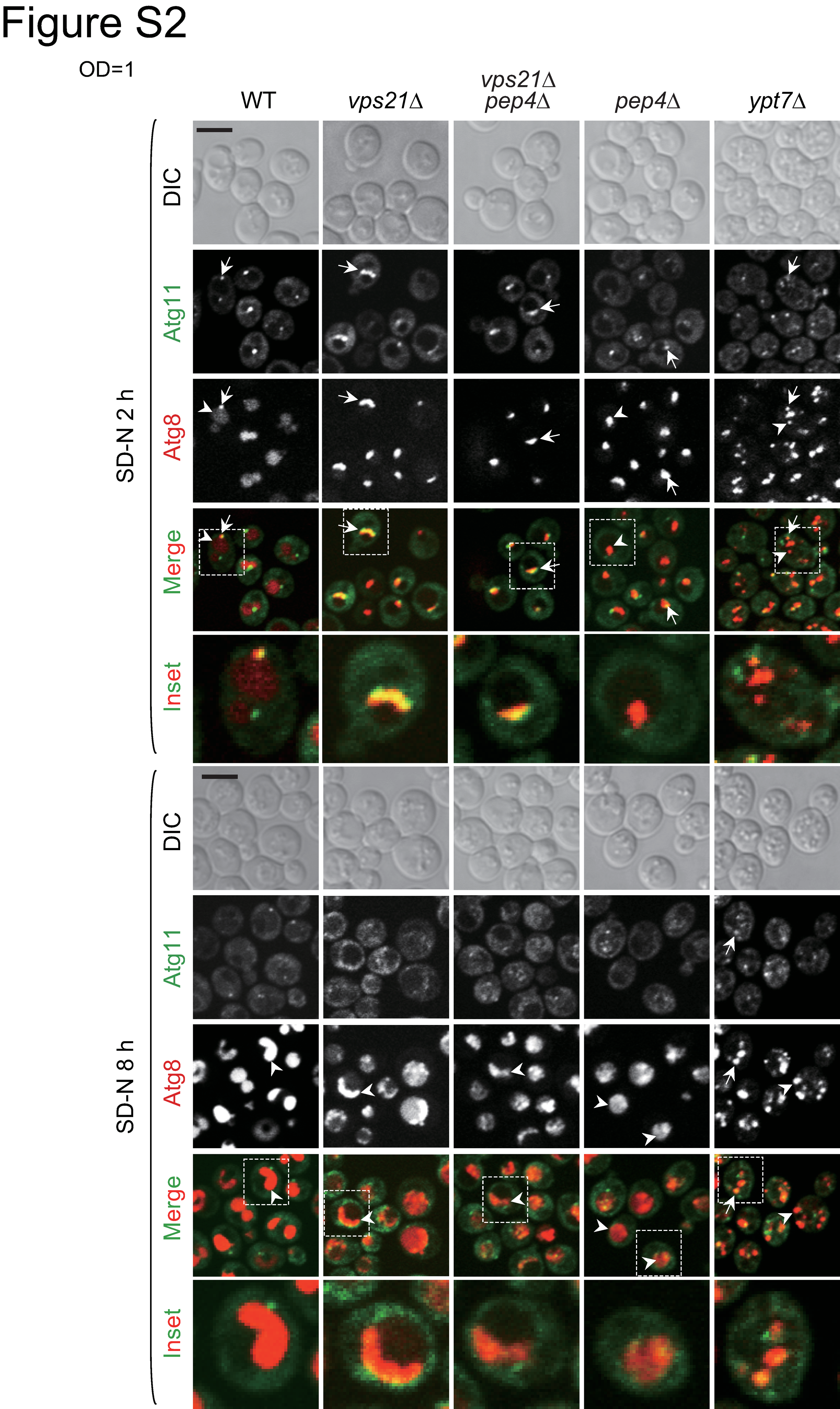

Supplement: S2 Fig — Strains expressing Atg11-GFP and mCherry-Atg8 from their chromosomes were grown and starved as reported [11] but with an additional nitrogen-starvation period of 8 h. Atg11-GFP colocalization with mCherry-Atg8 was monitored using live-cell fluorescence microscopy. The insets are from the frames for the merged pictures. The arrows indicate Atg11-Atg8 colocalization, and the arrowheads indicate cases where Atg8 did not colocalize with Atg11. Scale bars, 5 μm. Atg11 was removed from most Atg8-positive APs that accumulated in ypt7Δ cells before fusion or from most ABs that accumulated in pep4Δ cells after fusion, although Atg11 remained on most APCs on vacuole membranes that accumulated in vps21Δ cells after 2 h of nitrogen starvation (top and [10,11]). As APCs entered vacuoles in vps21Δ and vps21Δpep4Δ cells after 8 h of nitrogen starvation to become ABCs, most Atg11 detached from the ABCs. The results shown represent two independent experiments. (TIF) [file pgen.1010431.s002.tif]

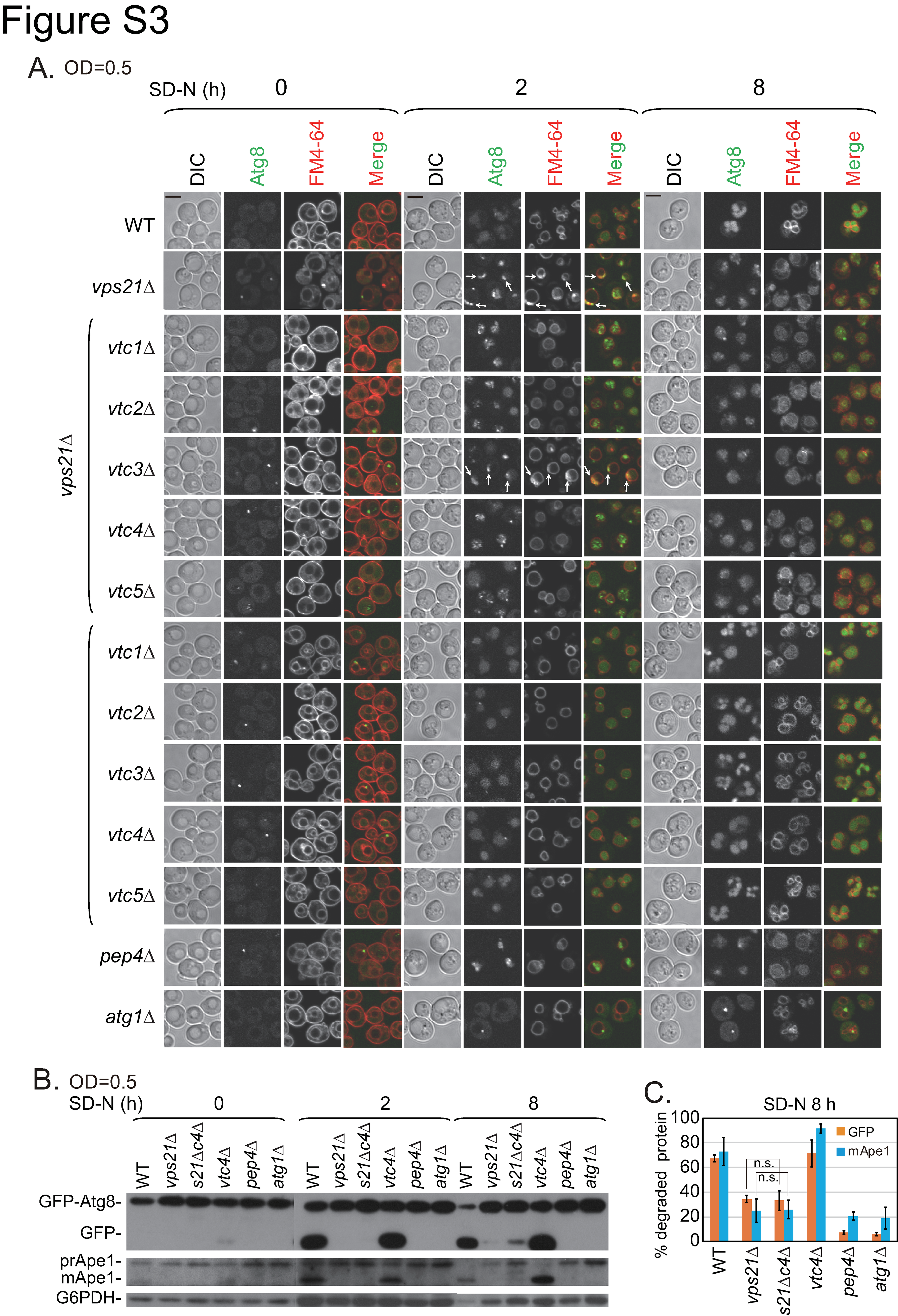

Supplement: S3 Fig — A. VTC1-5 were individually deleted from GFP-Atg8-labeled WT and vps21Δ cells to generate the indicated strains. The strains were grown and starved in SD-N medium as described in Fig 2A for microscopic observations. Except for Vtc3, the depletion of all other Vtc proteins promoted the entry of accumulated APCs into vacuoles in vps21Δ cells under nitrogen starvation. Scale bars at 5 μm; arrows, APCs. B. Immunoblotting assays showing that partial autophagy processing in vps21Δ cells after nitrogen starvation increased slightly with the depletion of Vtc4. Cells were grown as described in Fig 8A, and autophagy processing was determined as described in Fig 1D. C. The autophagy process (% GFP and % mApe1) was quantified after 8 h of SD-N treatment for the samples represented in panel B. The quantitative data are presented as the mean +/- STD. n.s., not significant. The results shown represent two independent experiments. (TIF) [file pgen.1010431.s003.tif]

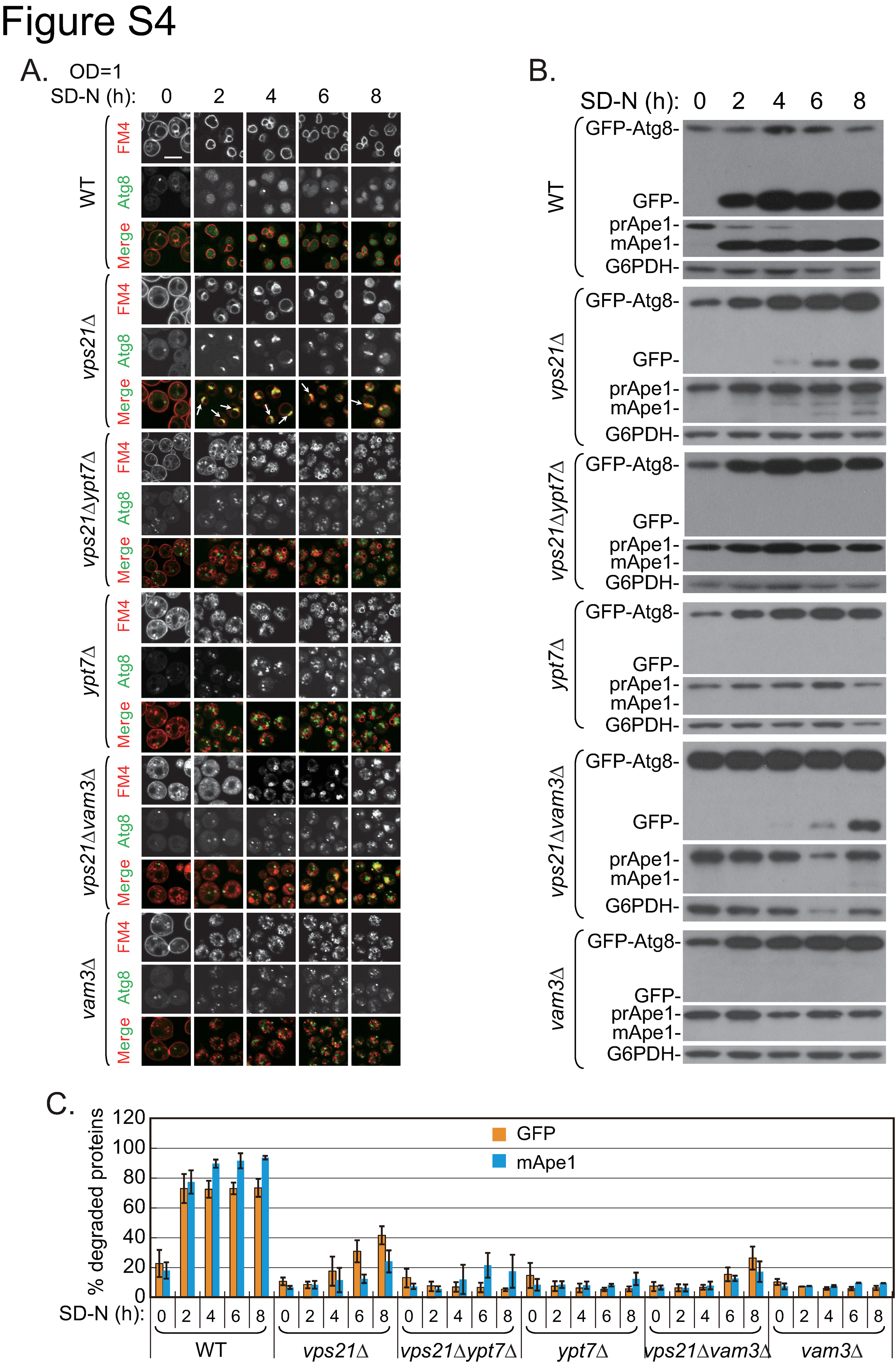

Supplement: S4 Fig — A. The entry of GFP-Atg8-labeled APCs into vacuoles was impaired when Ypt7 or Vam3 was depleted. YPT7 or VAM3 was deleted from vps21Δ cells to obtain vps21Δypt7Δ or vps21Δvam3Δ cells, respectively. The cells were grown, starved, and examined as described in Fig 2A. Arrows, APCs; scale bar, 5 μm. B. Autophagy processing was completely blocked in vps21Δypt7Δ cells and partially blocked in vps21Δvam3Δ cells after prolonged nitrogen starvation. The cells were grown as described in panel A. GFP-Atg8 and prApe1 processing were determined for cell lysates by performing immunoblotting assays as described in Fig 1B. C. Quantification of the blots presented in panel B. Quantification was performed as described in Fig 1D. The results shown represent at least two independent experiments. (TIF) [file pgen.1010431.s004.tif]

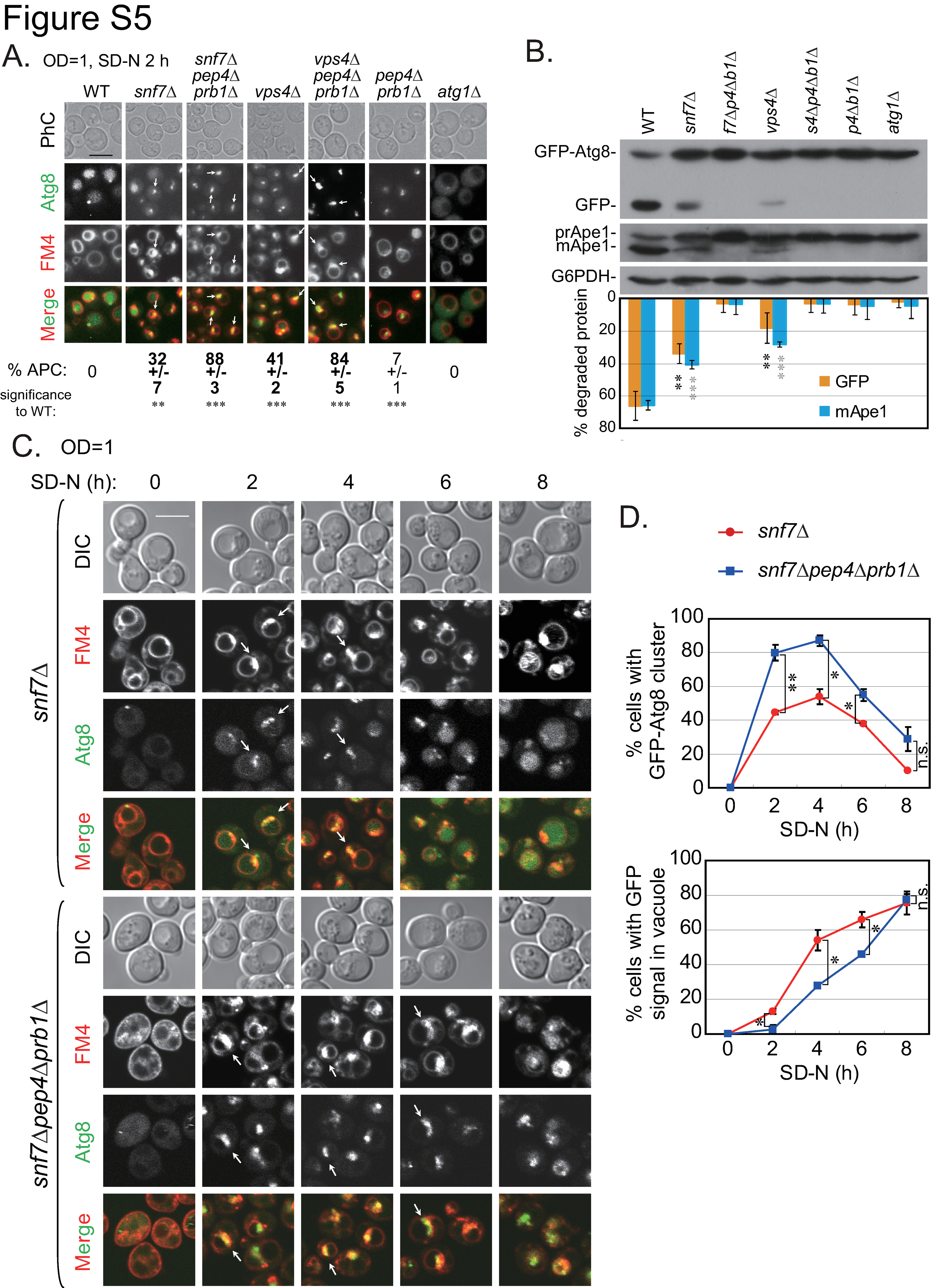

Supplement: S5 Fig — A. The accumulation of GFP-Atg8-labeled APCs in ESCRT-mutant (snf7Δ, vps4Δ) cells after nitrogen starvation increased in the absence of the vacuolar hydrolases Pep4 and Prb1. The cells were grown and starved as described in Fig 1E. The percentages of cells containing APCs were quantified and are presented below the merged pictures. B. Partial autophagy processing in ESCRT-mutant cells after nitrogen starvation was completely blocked in the absence of vacuolar hydrolases. The cells were grown as described in panel A and autophagy processing was determined and presented as described in Fig 1D. C. The accumulated APCs in snf7Δ and snf7Δpep4Δprb1Δ cells entered vacuoles after prolonged nitrogen starvation. The indicated cells expressing GFP-Atg8 (as shown in panel A) were grown and starved as described in Fig 2A for fluorescence observations. In panels A and C: arrows, APCs; scale bars, 5 μm. D. Quantification of the cells containing APCs and GFP signals in vacuoles shown in panel C. The percentage of cells containing APCs peaked after 4 h of nitrogen starvation and declined after that in snf7Δ and snf7Δpep4Δprb1Δ cells (top), whereas the percentage of cells containing GFP in vacuoles gradually increased in snf7Δ and snf7Δpep4Δprb1Δ cells (bottom) under prolonged nitrogen starvation. The quantitative data are presented as the mean +/- STD. *p < 0.05; **p < 0.01; n.s., not significant. The results shown represent at least two independent experiments. (TIF) [file pgen.1010431.s005.tif]

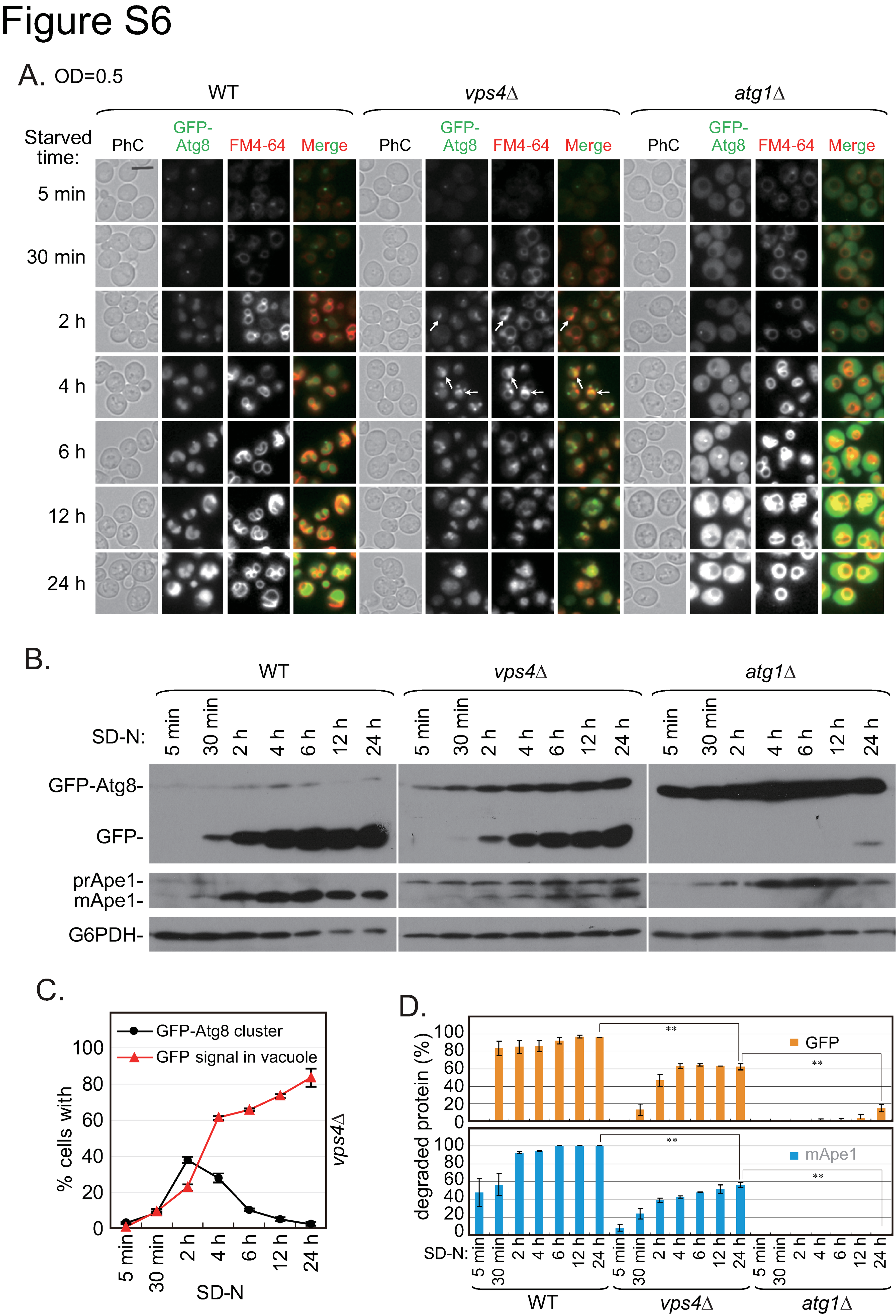

Supplement: S6 Fig — A. The accumulated GFP-Atg8-labeled APCs in vps4Δ cells gradually entered vacuoles after prolonged nitrogen starvation. The indicated cells that expressed GFP-Atg8 were grown and starved as described in Fig 2A for fluorescence observations. FM4-64 staining was performed to label the vacuole membranes for 1 h before the cells were collected for fluorescence microscopy. Arrows, APCs; scale bar, 5 μm. B. Autophagy processing in vps4Δ cells increased after prolonged nitrogen starvation. The cells were grown as described in panel A and subjected to immunoblotting assays as described in Fig 1B. G6PDH was detected as a loading control. C. Quantification of the vps4Δ cells shown in panel A that contained APCs and GFP signals in their vacuoles. The percentage of vps4Δ cells containing GFP in their vacuoles gradually increased, whereas the percentage of cells containing APCs peaked at 2h and subsequently declined after prolonged nitrogen starvation. D. The autophagy process was still defective in vps4Δ cells although prolonged nitrogen starvation partially promoted autophagy. The bands in panel B were quantified and presented as done in Fig 1F. The quantitative data are presented as the mean +/- STD. **p < 0.01. The results shown represent two independent experiments. (TIF) [file pgen.1010431.s006.tif]

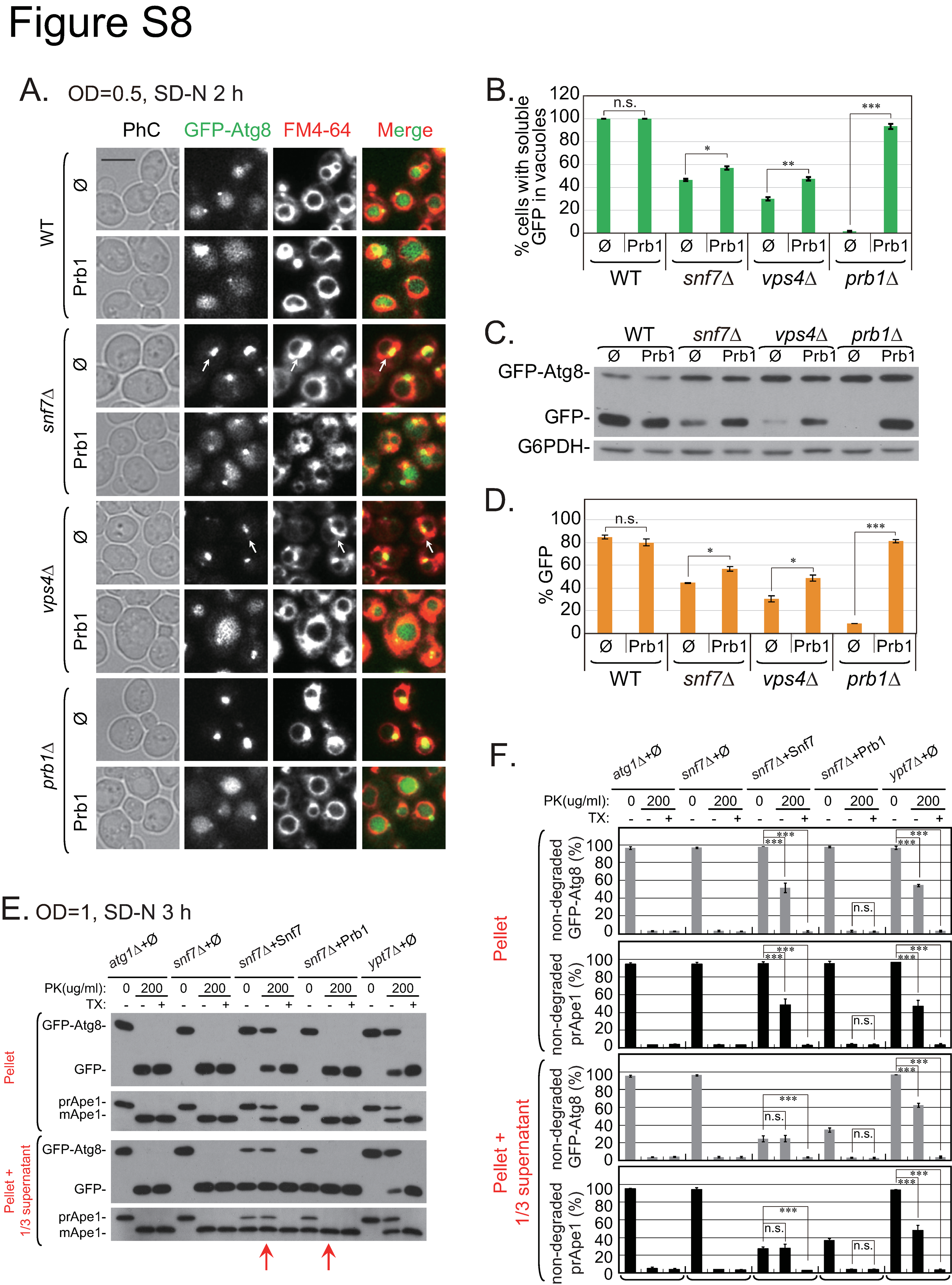

Supplement: S8 Fig — A. The accumulation of GFP-Atg8-labeled APCs in ESCRT- mutant (snf7Δ, vps4Δ) cells decreased with Prb1 overexpression. The indicated cells were transformed with a Prb1-expression plasmid or the empty vector (pRS415, ∅), grown, and examined as described in Fig 7A. Scale bar, 5 μm; arrows, APCs. B. Quantifications of cells containing soluble GFP in vacuoles. The percentage of cells containing GFP in vacuoles (shown in panel A) was quantified as the mean +/- STD. Over 600 cells were counted for each strain. C. GFP-Atg8 degradation in ESCRT- mutant cells increased with Prb1 overexpression. The cells were grown as described in panel A and examined for GFP-Atg8 degradation as described in Fig 1. G6PDH was detected as a loading control. D. Quantification of GFP-Atg8 degradation, based on the immunoblot shown in panel C. The quantification was performed as described in Fig 1D, and the data are presented as the mean +/- STD. E. The facilitation of autophagy by Prb1 in snf7Δ cells was not due to phagophore closure, as Snf7 did in snf7Δ cells. Cells transformed with a Prb1-expression plasmid, an Snf7-expression plasmid, or the empty vector (pRS415, ∅) were grown as described in panel A, except that they were starved for 3 h. The “pellet” and “pellet + 1/3 supernatant” sample sets were prepared from the indicated cells as described in the Materials and methods section and subjected to protease-protection assay and immunoblotting assays, exactly as described in Fig 6A and 6B. Either Prb1 or Snf7 in snf7Δ cells facilitated GFP-Atg8 degradation and prApe1 maturation (bottom), but only Snf7 (not Prb1) facilitated phagophore closure in snf7Δ cells. The red arrows point to these key results. F. The blots shown in panel E were quantified as described in Fig 6A and 6B. P values in panels B, D, and F: n.s., not significant; *p < 0.05; **p < 0.01; ***p < 0.001. The results shown represent at least two independent experiments. (TIF) [file pgen.1010431.s008.tif]

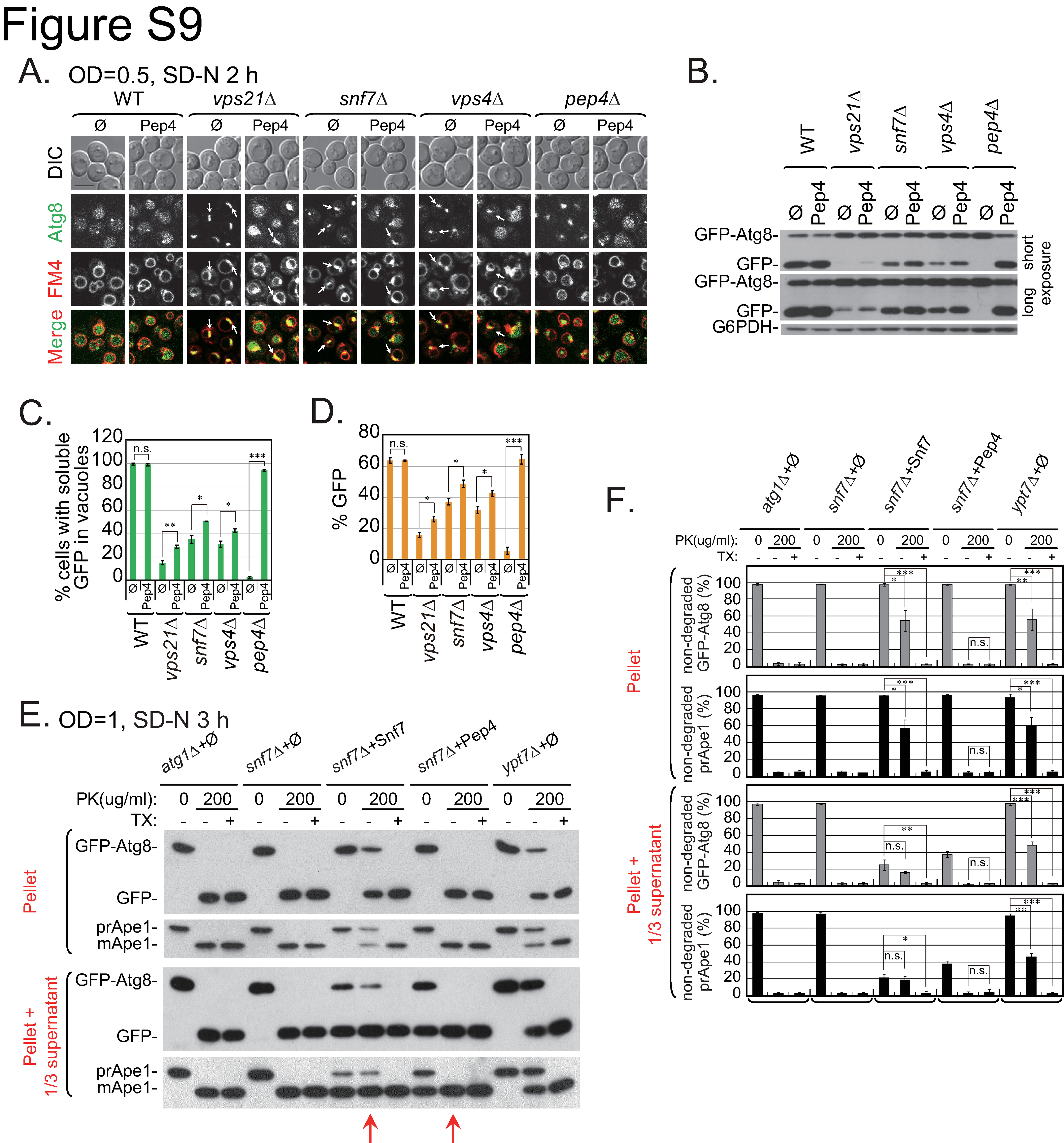

Supplement: S9 Fig — A. The accumulation of GFP-Atg8-labeled APCs in mutant cells (vps21Δ, snf7Δ, and vps4Δ) slightly decreased with Pep4 overexpression. The indicated cells were transformed with a Pep4-expression plasmid or the empty vector (pRS415, ∅), grown, and examined as described in Fig 7A. Scale bar, 5 μm; arrows, APCs. B. GFP-Atg8 degradation increased in Vps21- and ESCRT-mutant cells with Pep4 overexpression. The cells represented in panel A were examined for GFP-Atg8 degradation as described in Fig 1. C. Quantification of the cells shown in panel A that contained soluble GFP in their vacuoles, presented as the mean +/- STD. Over 250 cells were counted for each strain. D. Quantification of GFP-Atg8 degradation shown in the immunoblots in panel B. GFP-Atg8 degradation shown in panel B was quantified as described in Fig 1D, and the results are presented as the mean +/- STD. E. The facilitation of autophagy by Pep4 in snf7Δ cells was not due to phagophore closure, as Snf7 did in snf7Δ cells. Cells transformed with an Snf7-expression plasmid, a Pep4-expression plasmid, or the empty vector (pRS415, ∅) were grown as described in panel A, except that they were starved for 3 h. The experiments were conducted as described in S8E Fig. Either Pep4 or Snf7 in snf7Δ cells facilitated GFP-Atg8 degradation and prApe1 maturation (bottom), but only Snf7 (not Pep4) promoted phagophore closure in snf7Δ cells. The red arrows point to these key results. F. Quantification of the immunoblot results shown in panel E was performed as described in S8F Fig. P values in panels C, D, and F: n.s., not significant; *p < 0.05; **p < 0.01; ***p < 0.001. The results shown represent at least two independent experiments. (TIF) [file pgen.1010431.s009.tif]
