# Supplementary material for: The entry of unclosed autophagosomes into vacuoles and its physiological relevance
Source: PLoS Genet. 2022 Oct 13;18(10):e1010431. doi: 10.1371/journal.pgen.1010431 (PMC9562215; doi:10.1371/journal.pgen.1010431)
Supplement: S1 Table — A. Strains. B. Plasmids. (PDF) [file pgen.1010431.s010.pdf]

Table S1. Yeast strains and plasmids used in this study

A. Strains

Strain	Genotype	Source	Figures
YLY2422	SEY6210, *ura3::GFP-ATG8-URA3*	[1]	Fig. 1, 7-8, S3, S5A-B, S7A, S8A-D, S4, S6, S9A-D
YLY3086	YLY2422, *vps21Δ::LYS2*	[1]	Fig. 1-4, 6, 8, S1D, S3-4
YLY9511	YLY2422, *vps21Δ::LYS2 pep4Δ::hphMX4 prb1Δ::kanMX3*	This study	Fig. 1E-F, 2-4, 6, S1D,
YLY9561	YLY2422, *pep4Δ::hphMX4 prb1Δ::kanMX3*	This study	Fig. 1E-F, 2-4, 6, S1D, S5A-B, S7
YLY1735	SEY6210, *VPH1-GFP::HIS3MX6 ATG8::mCherry-ATG8-TRP1*	This study	Fig. 5
YLY1736	SEY6210, *VPH1-GFP::HIS3MX6 ATG8::mCherry-ATG8-TRP1 vps21Δ::hphMX4*	This study	Fig. 5
YLY3889	YLY2422,*ypt7Δ::hphMX4*	[1]	Fig. 6-7, S1D, S8E-F, S4, S9E-F
YLY5928	YLY2422, *atg1Δ::kanMX3*	[1]	Fig. 1E-F, S5A-B, S6
YLY6528	YLY2422, *atg1Δ::hphMX4*	This study	Fig. S8E-F, S9E-F
YLY5283	*Mat alpha leu2 trp1 ura3 lys2 ypt1-1 GFP-Atg8::URA3*	[2]	Fig. 7
YLY1628	YLY2422, *vps21Δ::kanMX3*	This study	Fig. 7, S1, S4, S9A-B
YLY9445	YLY2422, *prb1Δ::hphMX4*	This study	Fig. 7, S8A-D
YLY1848	YLY2422, *vps21Δ::LYS2 vtc4Δ::kanMX3*	This study	Fig. 8, S3
YLY1843	YLY2422, *vtc4Δ::kanMX3*	This study	Fig. 8, S3
YLY9853	SEY6210/SEY6210.1, *ura3::GFP-ATG8-URA3/ura3::GFP-ATG8-URA3*	This study	Fig. 9
YLY2674	SEY6210/SEY6210.1, *ura3::GFP-ATG8-URA3/ura3::GFP-ATG8-URA3 vps21Δ::kanMX3/vps21Δ::kanMX3*	This study	Fig. 9

YLY1504	SEY6210/SEY6210.1, *ura3::GFP-ATG8-URA3/ura3::GFP-ATG8-URA3 vps21Δ::kanMX3/vps21Δ::kanMX3 pep4Δ::hphMX4/pep4Δ::hphMX4*	This study	Fig. 9
YLY1505	SEY6210/SEY6210.1, *ura3::GFP-ATG8-URA3/ura3::GFP-ATG8-URA3 pep4Δ::hphMX4/pep4Δ::hphMX4*	This study	Fig. 9
YLY9854	SEY6210/SEY6210.1, *ura3::GFP-ATG8-URA3/ura3::GFP-ATG8-URA3 atg1Δ::kanMX3/atg1Δ::kanMX3*	This study	Fig. 9
YLY8470	SEY6210, *ATG8::mCherry-ATG8-TRP1 ATG11-3GFP::URA3*	[2]	Fig. S2
YLY8668	YLY8470, *vps21Δ::LYS2*	[2]	Fig. S2
YLY3437	YLY8470, *pep4Δ::hphMX4*	This study	Fig. S2
YLY3436	YLY8668, *pep4Δ::hphMX4*	This study	Fig. S2
YLY8610	YLY8470, *ypt7Δ::hphMX4*	[2]	Fig. S2
YLY1845	YLY2422, *vps21Δ::LYS2 vtc1Δ::kanMX3*	This study	Fig.S3
YLY1846	YLY2422, *vps21Δ::LYS2 vtc2Δ::kanMX3*	This study	Fig.S3
YLY1847	YLY2422, *vps21Δ::LYS2 vtc3Δ::kanMX3*	This study	Fig.S3
YLY1849	YLY2422, *vps21Δ::LYS2 vtc5Δ::kanMX3*	This study	Fig.S3
YLY1840	YLY2422, *vtc1Δ::kanMX3*	This study	Fig.S3
YLY1841	YLY2422, *vtc2Δ::kanMX3*	This study	Fig.S3
YLY1842	YLY2422, *vtc3Δ::kanMX3*	This study	Fig.S3
YLY1844	YLY2422, *vtc5Δ::kanMX3*	This study	Fig.S3
YLY1410	YLY2422, *vps21Δ::kanMX3 ypt7Δ::hphMX4*	This study	Fig. S4
YLY1412	YLY2422, *vps21Δ::kanMX3 vam3Δ::hphMX4*	This study	Fig. S4
YLY1411	YLY2422, *vam3Δ::hphMX4*	[1]	Fig. S4
YLY6405	YLY2422, *snf7Δ::kanMX3*	[3]	Fig. S5, S7-9

YLY9683	YLY2422, *snf7Δ::kanMX3* *pep4Δ::hphMX4 prb1Δ::TRP1*	This study	Fig. S5, S7
YLY6335	YLY2422, *vps4Δ::kanMX3*	[3]	Fig. S5A-B, S6-7, S8A-D, S9A-H
YLY9685	YLY2422, *vps4Δ::kanMX3* *pep4Δ::hphMX4 prb1Δ::TRP1*	This study	Fig. S5A-B, S7
YLY3195	YLY2422, *pep4Δ::hphMX4*	[1]	Fig. S9A-H

B. Plasmids

Plasmid	Alias	Genotype	Source
pYL893	pRS415	2µ, *LEU2,* Amp	This study
pYL1342		pRS415-*ADH1p-PEP4*	This study
pYL1343		pRS415-*ADH1p-PRB1*	This study
pYL125	pRS423	2µ, *HIS3*, Amp	[4]
pYL664		pRS423-*SNF7*	This study
pYL882	GFP	pFA6a-GFP-kanMX6	[5]
pYL1108	yEmCherry-Atg8	pRS304-yEmCherry-Atg8	[6]
pYL1252	Snf7-mCherry	ClhN-*SNF7-mCherry-TRP1*	This study
pYL609		pFA6a-GFP(S65T)-His3MX6	This study

References

1. Chen, Y., et al., *A Vps21 endocytic module regulates autophagy.* Mol Biol Cell, 2014. **25**(20): p. 3166-3177.
2. Zhou, F., et al., *A Rab5 GTPase module is important for autophagosome closure.* PLoS Genet, 2017. **13**(9): p. e1007020.
3. Zhou, F., et al., *Rab5-dependent autophagosome closure by ESCRT.* J Cell Biol, 2019. **218**(6): p. 1908-1927.
4. Sikorski, R.S. and P. Hieter, *A system of shuttle vectors and yeast host strains designed for efficient manipulation of DNA in Saccharomyces cerevisiae.* Genetics, 1989. **122**(1): p. 19-27.
5. Longtine, M.S., et al., *Additional modules for versatile and economical PCR-based gene deletion and modification in Saccharomyces cerevisiae.* Yeast, 1998. **14**(10): p. 953-61.
6. Graef, M., et al., *ER exit sites are physical and functional core autophagosome biogenesis components.* Mol Biol Cell, 2013. **24**(18): p. 2918-31.
